# Improved bioenergy value of residual rice straw by increased lipid levels from upregulation of fatty acid biosynthesis

**DOI:** 10.1186/s13068-023-02342-y

**Published:** 2023-05-27

**Authors:** Yunkai Jin, Jia Hu, Jun Su, Selcuk Aslan, Yan Lin, Lu Jin, Simon Isaksson, Chunlin Liu, Feng Wang, Anna Schnürer, Folke Sitbon, Per Hofvander, Chuanxin Sun

**Affiliations:** 1 Department of Plant Biology, The Linnean Centre for Plant Biology, Swedish University of Agricultural Sciences, P. O. Box 7080, 75007 Uppsala, Sweden; 2 Institute of Biotechnology, Fujian Academy of Agricultural Sciences, Fuzhou, 350003 China; 3 Hunan Provincial Key Laboratory of Crop Germplasm Innovation and Utilization, Hunan Agricultural University, Changsha, 410128 China; 4 Department of Molecular Sciences, Swedish University of Agricultural Sciences, P. O. Box 7015, 750 07 Uppsala, Sweden; 5 Department of Plant Breeding, Swedish University of Agricultural Sciences, P.O. Box 190, 23422 Lomma, Sweden

**Keywords:** WRINKLED1, Triacylglycerol (TAG), Rice, Endosperm, Bioenergy, Anaerobic digestion, Rice straw

## Abstract

**Background:**

Rice (*Oryza sativa*) straw is a common waste product that represents a considerable amount of bound energy. This energy can be used for biogas production, but the rate and level of methane produced from rice straw is still low. To investigate the potential for an increased biogas production from rice straw, we have here utilized WRINKLED1 (WRI1), a plant AP2/ERF transcription factor, to increase triacylglycerol (TAG) biosynthesis in rice plants. Two forms of *Arabidopsis thaliana* WRI1 were evaluated by transient expression and stable transformation of rice plants, and transgenic plants were analyzed both for TAG levels and biogas production from straw.

**Results:**

Both full-length AtWRI1, and a truncated form lacking the initial 141 amino acids (including the N-terminal AP2 domain), increased fatty acid and TAG levels in vegetative and reproductive tissues of Indica rice. The stimulatory effect of the truncated AtWRI1 was significantly lower than that of the full-length protein, suggesting a role for the deleted AP2 domain in WRI1 activity. Full-length AtWRI1 increased TAG levels also in Japonica rice, indicating a conserved effect of WRI1 in rice lipid biosynthesis. The bio-methane production from rice straw was 20% higher in transformants than in the wild type. Moreover, a higher producing rate and final yield of methane was obtained for rice straw compared with rice husks, suggesting positive links between methane production and a high amount of fatty acids.

**Conclusions:**

Our results suggest that heterologous WRI1 expression in transgenic plants can be used to improve the metabolic potential for bioenergy purposes, in particular methane production.

**Supplementary Information:**

The online version contains supplementary material available at 10.1186/s13068-023-02342-y.

## Background

The plant transcription factor WRINKLED 1 (WRI1) belongs to the APETALA2/ Ethylene Responsive Factor (AP2/ERF) superfamily, and contains two AP2 domains [1]. WRI1 binds with the AP2 domains to the AW-box ([CnTnG](n)_7_[CG]) located in the promoter region of downstream target genes, thereby regulating their expression [1–3]. Most of these target genes are important for carbon allocation into triacylglycerol (TAG) in plants [1]. The function of WRI1 was first reported in *Arabidopsis thaliana*, where the oil content in seeds of the *wri1* mutant was reduced by 80% [4]. Functional WRI1 orthologs have since then been identified in other plant species, including both monocots and dicots such as maize (*Zea mays*), wheat (*Avena sativa*), rapeseed (*Brassica napus*), potato (*Solanum tuberosum*), and yellow nutsedge (*Cyperus esculentus*) [3, 5–8]. Together these findings indicate a conserved importance of WRI1 in plant lipid metabolism. In addition to TAG synthesis, WRI1 also plays important roles in other biological processes, such as root development [9], flowering time regulation [10], and hormone signaling [11].

WRI1 is negatively autoregulated, and transient expression experiments in tobacco leaves have shown that the C-terminal region of WRI1 is important in the autoregulation process [12]. Based on a structural analysis, Ma et al. [13] showed that WRI1 has a total of three intrinsically disordered regions (IDRs); with one IDR being located upstream of the two AP2 domains, and two IDRs are located downstream. IDR3 has been shown to be important for the in vivo stability of Arabidopsis WRI1 through the presence of an internal PEST-motif [13].

TAG is the most important plant storage lipid, and is widely used for human consumption and other purposes [14–16]. The lipid yield of the top three crop species globally (maize, rice and wheat) is however limited (http://faostat.fao.org). This is in part due to the fact that the oil-rich parts; embryo and scutellum, only constitute a small part of a seed, usually less than 30% (v/v). The endosperm accounts for the main part of the seed, but is a starchy tissue with little TAG. Obviously, according to a cereal seed structure and oil content, increasing oil content in endosperm cells would be an efficient way to elevate oil production from cereals for food as well as bioenergy purposes.

Due to the climate effects of greenhouse gases, and the expected shortage of fossil fuels, it is necessary to develop renewable bioenergy sources. Increasing plant lipid production through metabolic engineering can be considered as an innovative platform to obtain energy and biofuel in more sustainable ways [17, 18]. In this context, rice straw represents a huge source of biomass that can be exploited to replace or reduce the dependency on fossil fuels [19]. Rice is the staple food in many countries, particularly in Asia. Rice straw constitutes ~ 50% of the gross weight of the paddy plant, and the production of rice straw from paddy fields is about 300–400 million tons in South Asia per year [20, 21]. The advantage of using rice straw to produce energy includes a continuous paddy production for human consumption [22]. Due to reasons of low cost, open burning is the most common method to retrieve energy from rice. However, this is considered undesirable because of a negative impact on the environment [19]. The energy content of rice straw is around 14 MJ per kg at 10% moisture content. Assuming 32% efficiency in gasification and combustion processes, 350,000 tons of rice straw might thus produce approximately 592,000 MWh of electricity [23]. The main methods currently implemented for power generation include direct combustion [24] and biogas production via anaerobic digestion (AD) [25–28]. Direct combustion is used because of its cost effectiveness. However, rice straw fuels have proved to be difficult to burn in most combustion furnaces, especially those designed for power generation, due to a high ash content (10–17%), and also a high content of silica, alkaline and chlorine components in the ash (https://www.bioenergyconsult.com). Rice straw has been evaluated in different studies for production of biogas; considered as the best route to exploit the energy in the straw [28]. Moreover, the digestate obtained from AD can be used as an organic fertilizer, as it contains plant available nutrient such as phosphorus and mineralized nitrogen [29]. However, the use of rice straw in practice is somewhat restricted due to high level of lignocellulose, a recalcitrant structure limiting microbial degradation, resulting in relatively slow degradation with low efficiency and biogas yields [28, 30], and the proportion of lignin in biomass has even been suggested to inversely proportional to the methane production [30]. AD of rice straw can be improved by various operational strategies, such as co-digestion with other materials giving a better nutrient balance, or by using a pre-treatment breaking up the recalcitrant structure [28, 31]. Another possible strategy to improve the efficiency and biogas yield, as investigated in the present study, is by regulating plant metabolism to reach a more optimal chemical composition.

The present study was conducted to investigate if WRI1 overexpression in transgenic rice plants can be used to increase the lipid (TAG and fatty acid) content, and thereby also improve biomethane production potential. To this end, a full-length *Arabidopsis thaliana* WRI1 cDNA, and a truncated form lacking the N-terminal AP2 domain, were overexpressed in transgenic rice plants.

## Results

### Transient expression of full-length and truncated versions of *AtWRI1* increases TAG levels in tobacco leaves

**Fig. 1:**
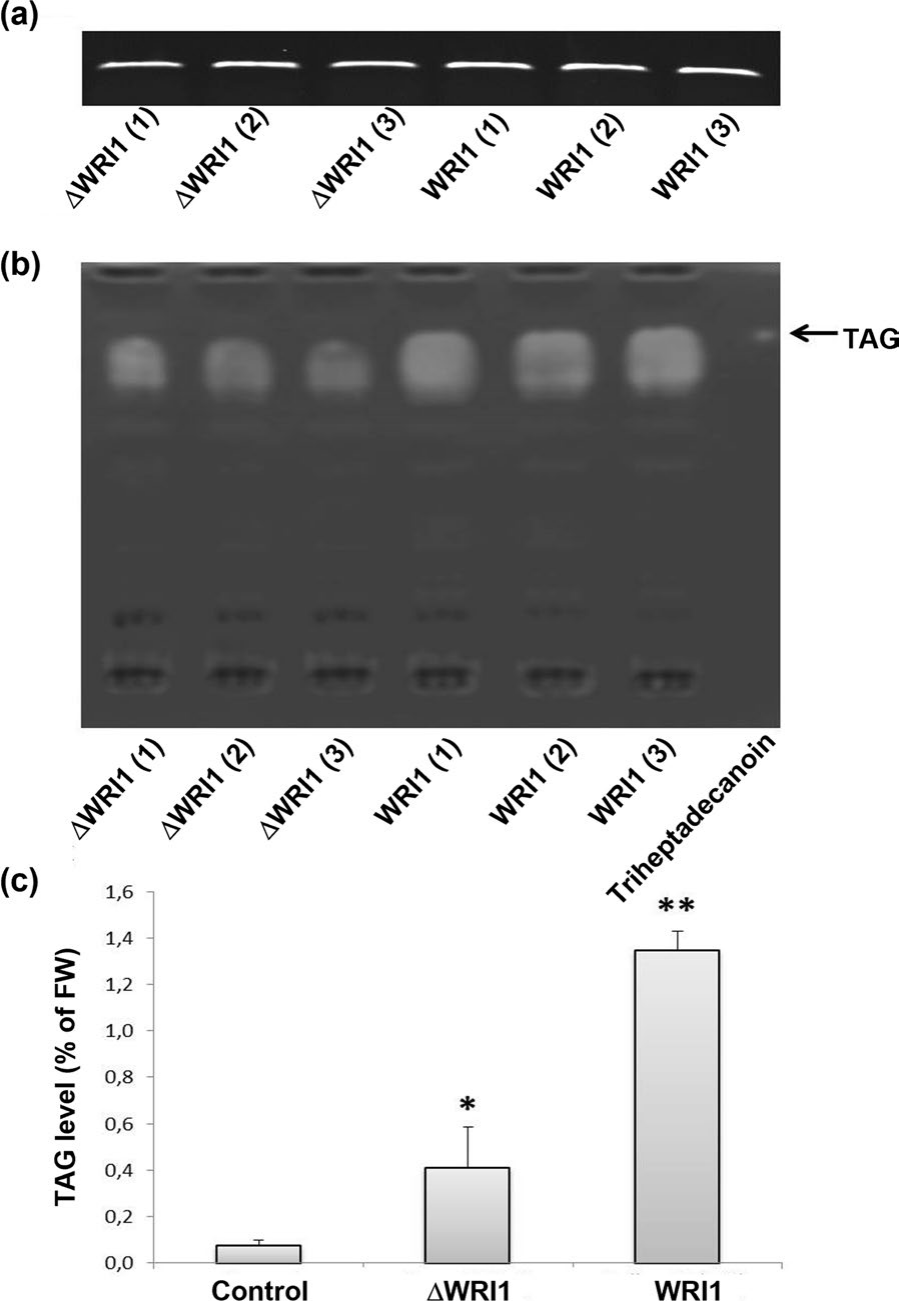
Activity of the full-length Arabidopsis *WRI1* (WRI1), and a truncated form (ΔWRI1) in tobacco leaves. **a** Semi-quantitative reverse-transcription PCR analysis of individual leaves after infiltration with p35S:*WRI1* or p35S:*ΔWRI1* strains. Numbers in parentheses indicate biological replicates. **b** Thin-layer chromatography analysis of triacylglycerols (TAG) in the infiltrated leaves. The position of TAG and the lipid reference triheptadecanoin is indicated by an arrow. **c** Quantification of total TAG levels in the infiltrated leaves using gas chromatography analysis. FW, fresh weight. Mean value ± SD from three biological replicates. Asterisks indicate a statistical difference compared to untreated control leaves, significant at *p ≤ 0.05 or **p ≤ 0.01 (Student´s *t*-test)

A full-length *Arabidopsis WRI1* cDNA, and a truncated form lacking the initial 114 amino acids including the first AP2 domain (*ΔWRI1*) (Additional file 1: Fig. S1), were expressed from the CaMV 35S promoter in a binary Ti-plasmid vector, and used for transient expression in tobacco (*Nicotiana benthamiana*) leaves. A high expression of both constructs was detected at five days after infiltration (Fig. 1a), and an increase of TAG oleo-chemicals was obvious at that time (Fig. 1b). Quantitative TAG analyses showed a 17-fold increase for p35S:*WRI1* compared to a control of none-infiltrated leaves, and that the TAG level was ca 1.4% on a fresh weight (FW) basis (Fig. 1c). Expression of p35S*: ΔWRI1* also led to increased TAG levels, but the degree of increase was significantly lower; ca. fivefold higher than controls (Fig. 1c), indicating that the N-terminal AP2 domain is important to WRI1 function and efficiency.

### Overexpression of *AtWRI1* and *ΔAtWRI1* in rice increases TAG levels in vegetative tissues

*AtWRI1* and *ΔAtWRI1* cDNAs were fused to the promoter of the barley starch branching enzyme IIb gene *HvSBEIIb* [32] to form two constructs: pSBE:*AtWRI1* and pSBE:*ΔAtWRI1*. The constructs were used to transform the Indica rice cultivar ‘Minghui 86’ (MH86). To compare the effect of *AtWRI1* expression in different rice varieties, we also transformed the pSBE:*AtWRI1* construct into Japonica rice cv. Nipponbare (Nipp); another agronomically important rice subspecies.

Integration of constructs in Indica rice was examined by Southern blot analysis (Additional file 1: Fig. S2). Analysis of TAG levels in three randomly chosen single-copy lines, showed that all lines contained significantly higher TAG levels than wild-type plants (Additional file 1: Fig. S3). In Indica rice, one line from each transformation was randomly selected for further phenotypic, genetic and metabolic characterizations during rice development. In Japonica rice, two independent lines were screened for further analyses.

**Fig. 2:**
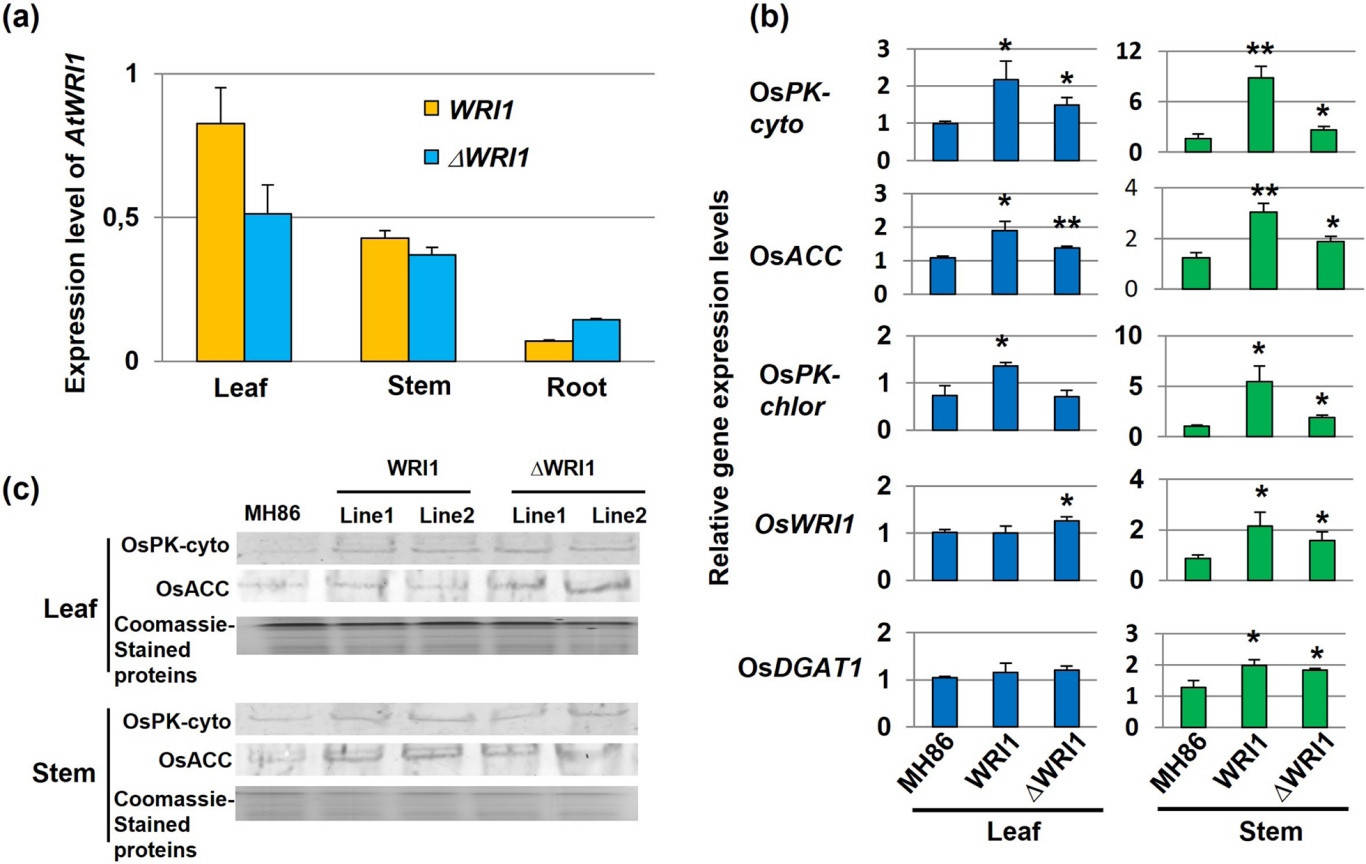
Effect of Arabidopsis *WRI1* overexpression on TAG levels in transgenic Indica rice plants. **a** Expression of full-length (*WRI1*) and truncated (*ΔWRI1*) *AtWRI1* transgenes from the barley pSBE promoter in vegetative plant tissues (leaf, stem, root). **b** Quantitative PCR analysis of the expression of five genes in fatty acid synthesis in WRI1 and ΔWRI1 transformants. Mean value ± SD from three biological replicates for each line. Asterisks indicate a statistical difference compared to the wild type (MH86), significant at *p ≤ 0.05 or **p ≤ 0.01 (Student´s *t*-test). **c** Western blot analysis of OsPK-cyto and OsACC proteins in leaf and stem of wild-type rice MH86 plants and two different lines for each construct. Quantification of signal intensities is shown in Additional file 1: Table S1

**Fig. 3:**
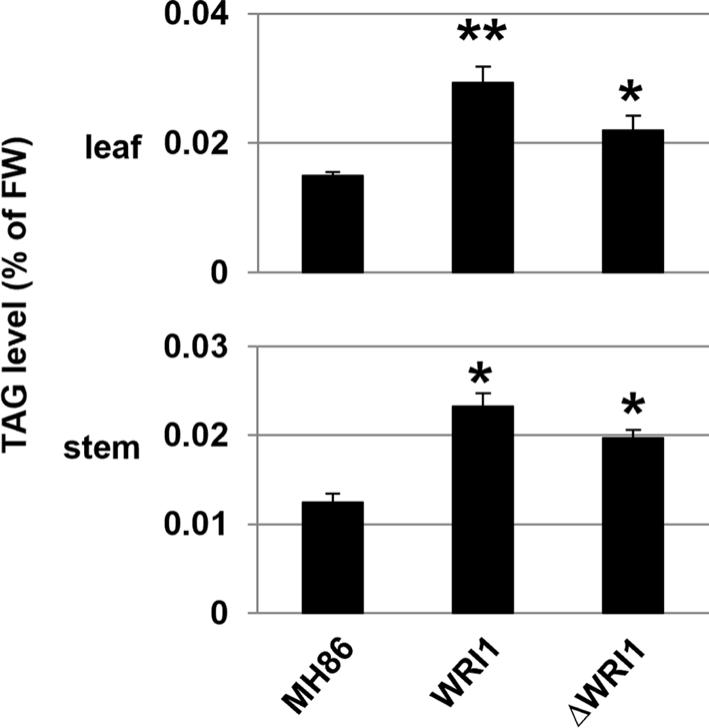
Fatty acid levels in vegetative tissues of wild-type Indica rice Minghui 86 (MH86) plants, and derived At*WRI1* transformants. Gas chromatography quantification of total TAG levels in leaves and stems from MH86, and WRI1 and ΔWRI1 transformants. Mean value ± SD from three biological replicates for each genotype. FW, fresh weight. WRI1: full-length *AtWRI1*; ΔWRI1: truncated *AtWRI1*. Asterisks indicate a statistical difference compared to the wild type, significant at *p ≤ 0.05 or **p ≤ 0.01 (Student´s *t*-test)

Phenotypic characterization was based on analyses of panicle length, thousand seed weight, tiller number, plant height and filled grains per panicle. The results indicated that the expression of *AtWRI1* in both Indica and Japonica rice did not change any of these traits (Additional file 1: Figs. S4, S5). Genetic and metabolic characterizations were performed in vegetative tissues (i.e., leaf, stem and root) sampled at 8 weeks from germination in Indica rice and at different developmental stages in Japonica rice. Quantitative PCR (qRT-PCR) analysis in Indica rice showed higher expression levels of both *AtWRI1* and *ΔAtWRI1* in above-ground tissues, especially in leaves (Fig. 2a). Further analyses showed that the expression of four genes in fatty acid synthesis: pyruvate kinase cytosolic isozyme (*OsPK-cyto*)*,* pyruvate kinase isozyme g chloroplast (*OsPK-chlor*), acetyl-CoA carboxylase (*OsACC*)*,* diacylglycerol transferase 1 (*OsDGAT1*), as well as *OsWRI1*, was generally higher in both Indica and Japonica transformants than in the corresponding wild type. This was particularly true for *OsPK-cyto, OsACC, OsPK-chlor*, which were significantly more expressed in stems and leaves from the different transformants (Fig. 2b, Additional file 1: Fig. S6a). In order to monitor the corresponding protein levels, we generated antibodies against OsPK-cyto (E.C. 2.7.1.40) and OsACC (E.C.6.4.1.2), corresponding to 15 and 13 amino acids in the protein sequence, respectively. Results showed that both WRI1 and ΔWRI1 rice had higher protein levels of OsPK-cyto and OsACC in leaf and stem compared with wild type (Fig. 2c, Additional file 1: Fig. S6b, Table S1). As expected from the gene expression analyses, WRI1 lines from both Indica and Japonica rice had significantly higher TAG levels compared to corresponding wild-type plants (Fig. 3, Additional file 1: Fig. S7). The TAG increase in both leaves and stems was greater in WRI1 transformants than in *Δ*WRI1 ones, being ~ twofold in WRI1 and ~ 1.5-fold in ΔWRI1 (Fig. 3). Taken together, the results indicate that *AtWRI1* can increase TAG levels in vegetative tissues of both Indica and Japonica rice, and that *AtWRI1* is somewhat more efficient than *ΔAtWRI1* in this aspect.

### Both *AtWRI1* and *ΔAtWRI1* increase TAG levels in rice seeds

**Fig. 4:**
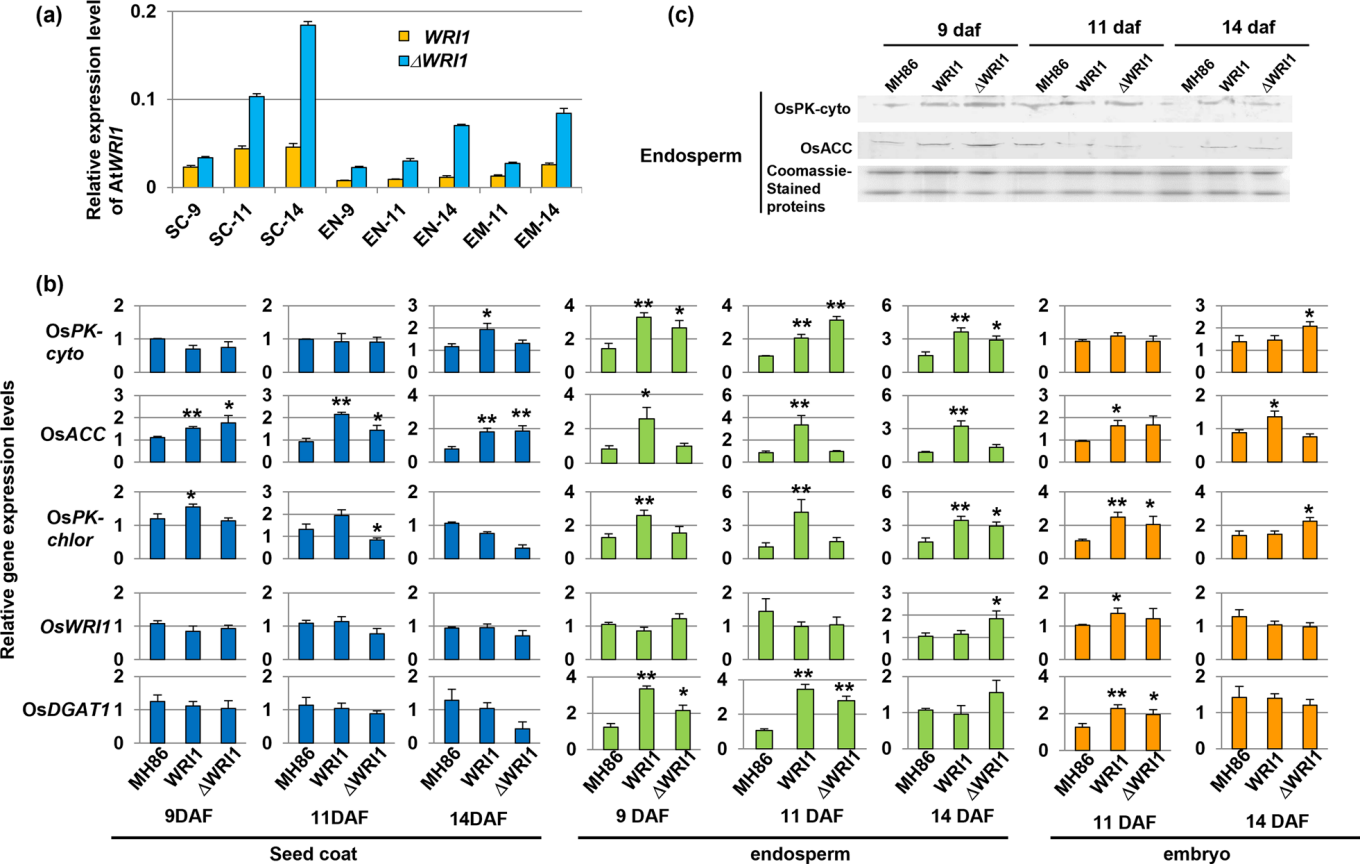
TAG levels in reproductive tissues of wild-type Indica rice Minghui 86 (MH86), and derived At*WRI1* transformants. **a** Relative expression of *WRI1* and ∆*WRI1* transgenes in the seed coat (SC), endosperm (EN) and embryo (EM). **b** Relative expression of genes in fatty acid synthesis, as analyzed in the seed coat, endosperm, and embryo. Mean value ± SD from three biological replicates for each genotype. Asterisks indicate a statistical difference compared to the wild type, significant at *p ≤ 0.05 or **p ≤ 0.01 (Student´s *t*-test). **c** Western blot analysis of OsPK-cyto and OsACC protein levels in leaf and stem. Quantifications of signal intensities are shown in Additional file 1: Table S1. daf: day after fertilization; WRI1: full-length *AtWRI1*; ΔWRI1: truncated *AtWRI1*

Indica rice seeds were sampled at different developmental stages (9, 11 and 14 days after fertilization), and dissected into seed coat, endosperm and embryo. For both the pSBE:*AtWRI1* and pSBE:*ΔAtWRI1* constructs, the relative expression was highest in the seed coat, and for the seed sections analyzed also highest at 14 days after fertilization (Fig. 4a). This is in good agreement with the native *SBEIIb* promoter activity as reported in barley [32]. The higher expression level of *AtWRI1* in the seed coat might indicate a more active carbohydrate metabolism in that tissue, e.g., to supply enough nutrition for endosperm and embryo development.

The expression of fatty acid biosynthesis genes in Indica rice was altered by expression of the two transgenes in the seed. For instance, *OsPK-cyto*, *OsACC, OsPK-chlor* and *OsDGAT1* were upregulated in most seed sections, particularly in the endosperm and embryo (Fig. 4b). By contrast, expression of native *OsWRI1* was significantly altered only in a few situations and to a lesser degree compared to the other genes (Fig. 4b). In line with the higher expression of *OsPK-cyto* and *OsACC*, Western blot analysis showed that the corresponding protein levels of OsPK-cyto and OsACC were increased (Fig. 4c, Table. S1). A similar molecular analysis was performed also in Japonica rice lines; at 9 daf and 14 daf in endosperm, or whole seeds. Consistent with the results from Indica rice, *OsPK-cyto*, *OsACC*, *OsPK-chlor* and *OsDGAT1* were all upregulated by the overexpression of *AtWRI1* (Additional file 1: Fig. S8a). Likewise, Western blot analysis showed that the expression of AtWRI1 in Japonica rice increased the protein levels of OsPK-cyto and OsACC (Additional file 1: Fig. S8b, Table S1).

**Fig. 5:**
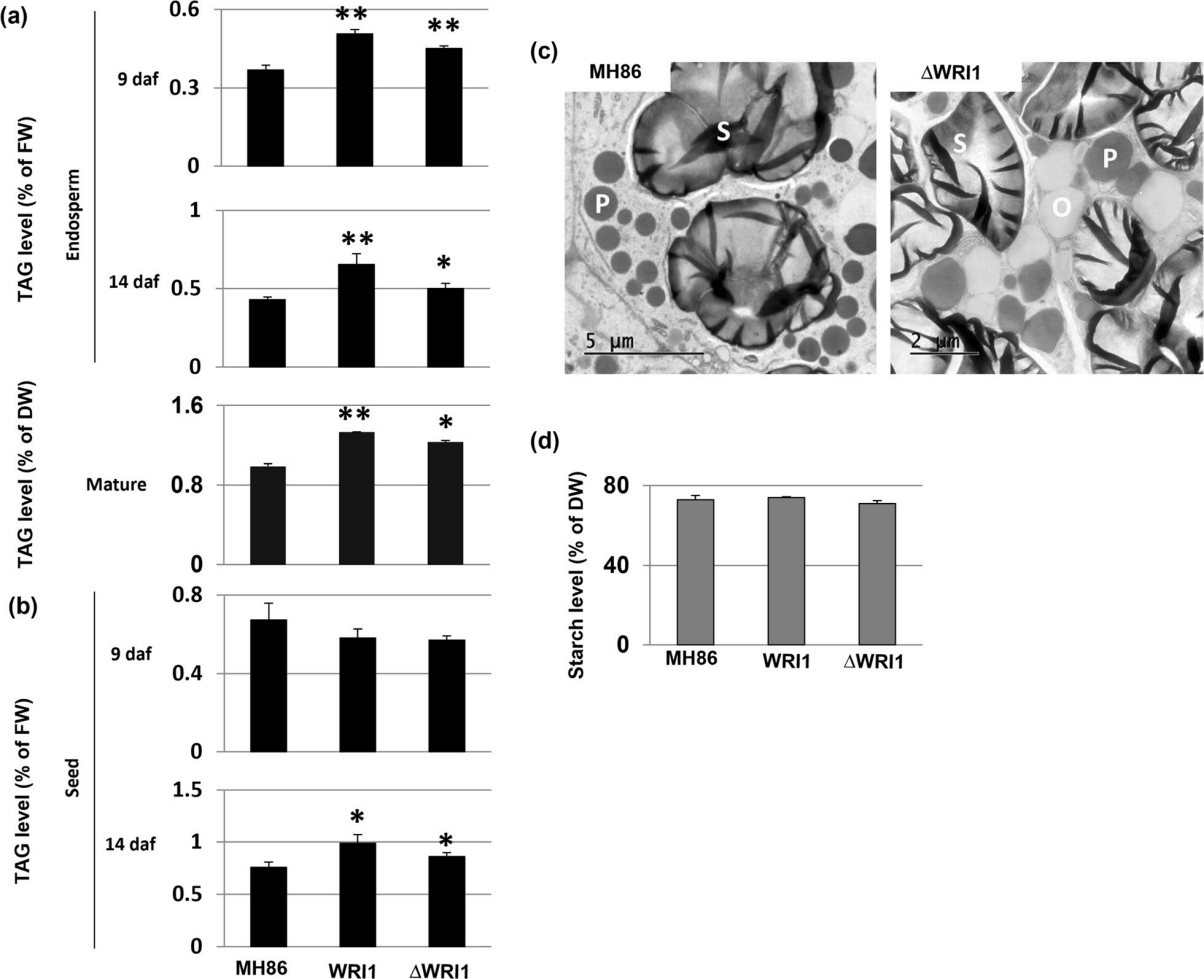
Fatty acid and starch levels in reproductive tissues of wild-type Indica rice Minghui 86 (MH86) plants, and derived At*WRI1* transformants. **a** Quantification by gas chromatography analysis of TAG level in endosperm, and in mature seeds without embryo. **b** TAG level in whole seeds at different times after fertilization (daf). **c** Transmission electron microscopy analysis of endosperm structures in MH86 and a transgenic line (ΔWRI1). S, starch; P, proteins; O, oil body. Scale bars (5 µm or 2 µm) are indicated. **d** Starch level in mature seeds without embryo. Mean value ± SD from three biological replicates for each genotype. FW, fresh weight; DW, dry weight; daf: day after fertilization; WRI1: full-length *AtWRI1*; ΔWRI1: truncated *AtWRI1*. Asterisks indicate a statistical difference compared to wild-type control plants, significant at *p ≤ 0.05 or **p ≤ 0.01 (Student´s *t*-test)

Because of the sample limitation, TAG levels could be measured only in endosperm and entire seeds. Both *AtWRI1* and *ΔAtWRI1* increased TAG levels in endosperm at 9 daf, 14 daf, as well as in mature seeds where the embryo had been removed (Fig. 5a, Additional file 1: Fig. S9a). In the intact seed, increased TAG levels were detectable only at 14 daf (Fig. 5b, Additional file 1: Fig. S9b). In the *ΔAtWRI1* lines, a number of oil bodies in the 14 daf endosperm cells were visible by electron microscopy, but not in MH86 (Fig. 5c). Starch is a main reserve product in rice seed, and in order to study a possible effect of *AtWRI1* on starch synthesis, we also analyzed the starch content in mature seed without embryo. The result showed no significant alterations of starch levels (Fig. 5d, Additional file 1: Fig. S9c), indicating that the expression of *AtWRI1* does not affect starch synthesis in any of the two rice varieties. The nitrogen content in husk-removed rice grain was similar between wild-type and WRI1 transformants (Additional file 1: Fig. S10), indicating that the WRI1 expression did not infer with this aspect of seed quality.

### Expression of *AtWRI1* improves chemical composition of rice straw for bioenergy purpose

**Fig. 6:**
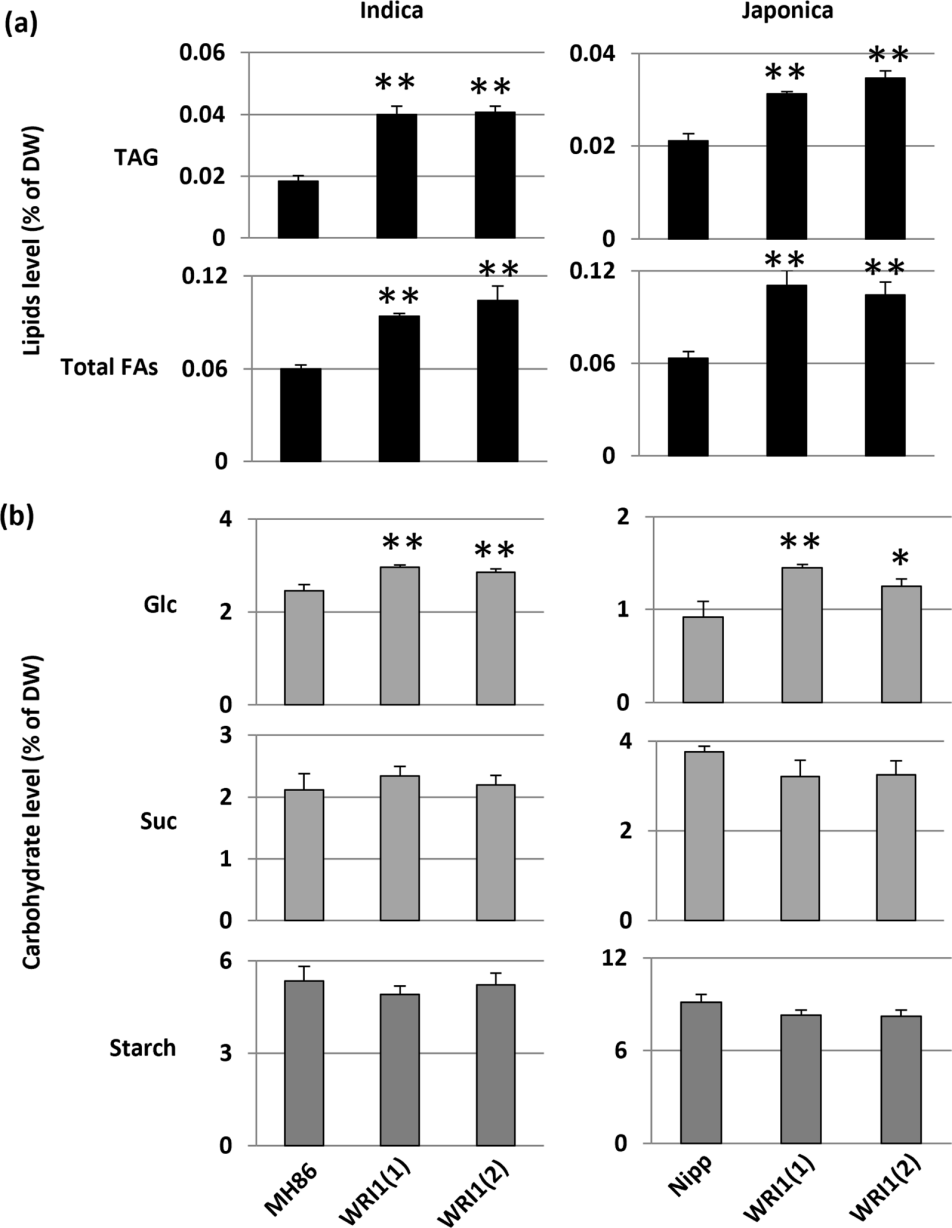
Fatty acid and carbohydrate levels in rice straw. **a** Quantification by gas chromatography analysis of total triacylglycerol (TAG) and total fatty acids (FAs) levels in wild-type rice plants and two transgenic lines for both Indica rice Minghui 86 (MH86) and Japonica rice Nipponbare (Nipp), expressing the same full-length AtWR1 construct (WRI1). **b** Glucose (Glc), sucrose (Suc), and starch, levels in the same genotypes. WRI1(1) and WRI1(2) indicate different transgenic lines. Mean value ± SD from three biological replicates for each genotype. DW: dry weight. Asterisks indicate a statistical difference compared to wild-type control plants, significant at *p ≤ 0.05 or **p ≤ 0.01 (Student´s *t*-test)

After grain harvest, the straw (vegetative parts) becomes a by-product, representing a large quantity of non-food biomass. The straw can be used for forage or as a soil improver, but its chemical energy can also be harvested. Traditionally this is done by burning, but more sustainable ways for bioenergy generation are gaining increased interest and are becoming more frequent. To investigate the potential for bioenergy purposes, we analyzed the lipid composition of the straw. This showed significantly higher TAG levels compared with wild type, being ca twofold higher in both Indica and Japonica varieties (Fig. 6a). The levels of total fatty acids were also significantly increased (Fig. 6a). Because AtWRI1 is part of the regulation of carbon allocation into fatty acids in plants [1], we also analyzed carbohydrate levels, *e.g.,* glucose, sucrose and starch, in the rice straw. Results showed that the level of glucose in transgenic plants was significantly increased in both Indica and Japonica rice, whereas sucrose and starch content was not different compared to controls (Fig. 6b). This indicates that the increased glucose did not become used for starch and sucrose production. Compared to Indica rice, straw from wild-type Japonica rice contained less glucose, but more starch and sucrose (Fig. 6b). The total organic carbon content in straw was similar in WRI1 and MH86 plants (Additional file 1: Fig. S11a).

### Expression of *AtWRI1* increases bioenergy production from rice straw

**Fig. 7:**
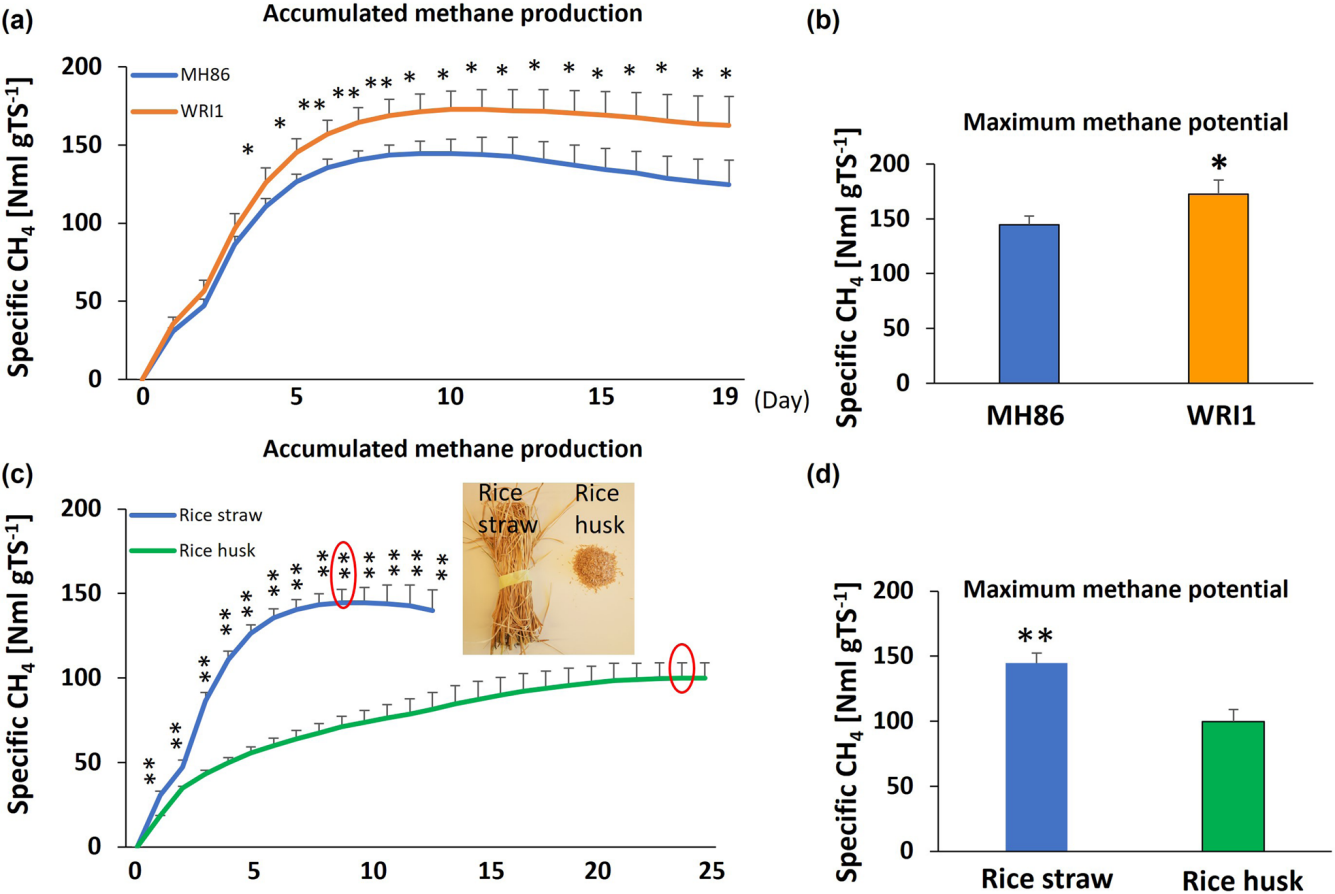
Anaerobic digestion assay of Indica rice straw and rice husk. **a** Accumulated methane production from day 0 to day 19, in wild-type Minghui 86 (MH86) rice plants, and derived WRI1 transformants. **b** Maximum methane potential values for MH86, and WRI1 transformants. **c** Accumulated methane production from day 0 to day 13 (rice straw) and from day 0 to day 25 (rice husk), maximum methane collection values are marked with red circles. **d** Maximum methane potential values for rice straw and rice husks. Nml: Normal ml (normalized to 1.01325 bar and 273.2 K); TS, Total solids. Mean value ± SD from three biological replicates for MH86, and of five replicates of two lines for WRI1 rice. Indica rice was used to collect rice straw and rice husks for C and D. Asterisks indicate a statistical difference compared to the wild type, significant at *p ≤ 0.05 or **p ≤ 0.01 (Student´s *t*-test)

The higher levels of fatty acids and glucose in transgenic rice straw suggested that bioenergy production from straw can be increased. To investigate this possibility, straw from Indica rice was used as substrate for biogas production during anaerobic digestion (AD). Methane analyses showed that WRI1 rice straw produced significantly more methane than the wild type. This difference was obvious from day 4 and to the end of the experiment (Fig. 7a). The maximum methane collection in WRI1 rice was significantly higher than in the wild-type control (173 vs 145 ml/g TS of rice straw); i.e., a close to 20% increase (Fig. 7b). By contrast, there were no significant differences between the two genotypes with regard to their straw combustion values (Additional file 1: Fig. S11b). An overview of biomethane yield from different materials and their oil, carbohydrate and total organic carbon contents is given in Additional file 1: Table S2.

To evaluate the full bioenergy production potential of the rice residuals, the methane production potential was determined also for the rice husk. This showed lower methane production rates for the husk as compared to the straw (Fig. 7c). Moreover, the maximum methane production rate from straw was observed at day 9, but at day 23 for rice husks (Fig. 7c). The final methane production value from straw was significantly higher than from the husks, reaching 145 ml/g (Fig. 7d).

## Discussion

Efforts to increase oil content using WRI1 overexpression in transgenic cereal seeds have previously been made in maize, where the seed oil content in transgenic WRI1 plants was elevated due a TAG increase in embryos, but not in the endosperm [6, 33, 34]. Here, we show in transgenic Indica and Japonica rice plants that the endosperm oil content can be significantly increased by overexpression of both full-length and truncated versions of the Arabidopsis *WRI1* gene (Fig. 5 and Additional file 1: Fig. S9).

A TAG increase in the endosperm of our WRI1-overexpressing rice plants is supported by the fact that endosperm tissues were carefully detached from the rice seeds, and that high TAG levels were shown in these tissues (Fig. 5 and Additional file 1: Fig. S9). In addition, the increased numbers of oil bodies in the ΔWRI1 rice endosperm (Fig. 5c) also supports this conclusion. It is not clear why WRI1 overexpression leads to increased oil content in rice endosperm but not in maize. Possible explanations could be that the difference reflects a homologous *ZmWRI1* overexpression in maize [6, 33], but a heterologous *AtWRI1* expression in rice. It is also possible that the maize and Arabidopsis WRI1 proteins function differently in the endosperm, or that the use of different promoters leads to differences in tissue-specific transgene expression. In rice, two endogenous *OsWRI1* genes and two additional homologs were reported [35], but their precise function in lipid synthesis is unknown. The barley-derived *SBEIIb* promoter senses photosynthate [32], and the expression of *AtWRI1* from the *HvSBEIIb* promoter can increase the carbon flux from photosynthate to fatty acids in both vegetative and reproductive tissues.

The IDR3 part from the WRI1 C-terminal region was shown to be important for the in vivo stability of *Arabidopsis* WRI1 through the presence of a PEST-motif [12]. Little is known about the relationship between the structure and function for the plant AP2 superfamily transcription factors, e.g., why the family members possess one or two AP2 domains [36, 37]. Our results show that full-length and truncated WRI1 have different abilities to activate target gene expression and enhance TAG synthesis in both tobacco leaves and in Indica rice (Figs. 1–5). It is unclear how many downstream genes that are regulated by the AtWRI1 protein in rice, but we showed that *OsACC*, *OsPk-chlor* and *OsPk-cyto* were all upregulated and well correlated to the overexpression of *AtWRI1* (Figs. 2, 4, Additional file 1: Fig. S6, S8), indicating that they are indeed regulated by AtWRI1. Results showing that all four fatty acid genes investigated could be activated in vivo by both full-length and truncated constructs, and that *ΔAtWRI1* led to a weaker response than *AtWRI1* in both types of transformant as well as in the transient assay, suggest that the first AP2 domain is more important for the degree of transcription activity than for the target gene specificity.

Rice straw is a biomaterial with the potential to replace or reduce the dependency on fossil fuels. Important advantages are a low cost and a huge area of global production [38]. We speculated that the increased levels of lipids, TAG and glucose in WRI1 rice straw might provide increased levels of easily available carbon sources and with increased methane potential. The increase of glucose could also bypass the hydrolytic step and lead to an increased methane production rate and, as lipids has a high methane potential, improve the methane yield [39]. Indeed, the methane production was increased by close to 20% in the transformants, in line with the increased content of lipids. However, no difference in the rate of methane production was observed, suggesting that the hydrolytic step still was rate limiting. The biomethane production of raw rice straw without any pre-treatment has been reported to be ca 135 L/kg VS [30]. Given that VS (organic matter) represents 95.26% of TS (total solid) content, 135 L/kg VS biomethane corresponds to 142 ml/g TS, well in line with the methane production value: 145 ml/g TS, from wild-type rice straw in this paper. The production of 173 ml/g TS methane from WRI1 rice straw thus represents a ca 20% increase also in comparison to the previous study [30]. Also, the use of pre-treatment can significantly enhance the methane production from straw, but this might be considered disadvantageous for economic and environmental reasons [25–28]. In this regard the transgenic rice represents an interesting and cheaper option to improve methane yields from rice straw.

The difference in methane production was clearly linked to the fatty acid contents, as only small differences were seen in the carbohydrate and total organic carbon contents between WRI1 rice and WH86 (Additional file 1: Table S2). The higher rate, and higher amount, of methane production from rice straw compared with rice husks was expected, and was most probably also related to the difference in fatty acid content. As reported, the fatty acid content in rice straw is more than two times higher than in rice husks [40, 41]. The fact that the total organic carbon and combustion energy values did not differ between wild-type and transformants, indicates that the observed increase in methane production is related to the metabolic composition of the straw, rather than to the amount of fixed carbon as such from photosynthesis. The latter is supported by our phenotypic analyses showing that expression of *AtWRI1* in both Indica and Japonica rice did not affect yield or any other measured physiological trait compared to wild-type plants (Additional file 1: Figs. S4, S5). In addition, the similar total nitrogen content in husk-removed rice grain indicates the expression of WRI1 also did not affect protein level in seed (Additional file 1: Fig. S10). At present we do not know the relative importance of increased levels of fatty acids vs glucose for the methane production. Nonetheless, our study demonstrates that transgenic plants may become useful models for more sustainable uses of rice by-products with regard to bioenergy production methods.

## Conclusions

The results presented here demonstrate that both full-length and truncated *AtWRI1*, when expressed from the *HvSBEIIb* promoter, can increase lipid biosynthesis in transgenic rice plants. A lower stimulatory effect of the truncated AtWRI1 form than of the full-length protein, suggests a role for the deleted AP2 domain in WRI1 activity. Expression of *AtWRI1* in vegetative tissues increases lipid levels in rice straw, and is associated with a 20% higher methane production from anaerobic digestion. Collectively our results suggest that heterologous *WRI1* expression in transgenic plants can be used to improve the metabolic potential for bioenergy purposes, in particular methane production.

## Materials and methods

### Plant materials and growth conditions

*Nicotiana benthamiana* plants were grown in cylinder-type pots (22 cm high with an upper diameter of 25 cm and bottom diameter of 19 cm, and filled with soil). The plants were grown in a phytotron unit under conditions of 16 h light/8 h dark, a constant temperature of 25 °C, and a relative humidity of 60%. The photon flux was 320 µmol m^−2^ s^−1^. The plants were watered regularly with commercial fertilizer according to common practice [42]. The top two leaves of 6-week-old plants were used for infiltration, and the leaf samples were harvested five days after infiltration for different analyses.

Rice (*Oryza sativa L.*) plants were grown in pots (30 cm high with an upper diameter of 29 cm and bottom diameter of 19 cm) in phytotron unit. The phytotron conditions were set as 14 h light/10 h dark with 30 °C/25 °C. The relative humidity was 80% and the photon flux was 400 µmol m^−2^ s^−1^ [43]. The plants were watered with 1% of N.P.K macronutrients [42] at tilling stages and flowering stage and with pure water at other stages.

For analyses of chemical composition, rice straw from the mature stage was harvested starting at 3:00 pm, dried at 70 °C for 3 days, and ground into fine powder at room temperature using a Tissuelyser.

### Vector construction, leaf transient expression, and transformation of rice plants

Nucleotides No. 344–1414 (full-length open reading frame; *WRI1*) and 373–1414 (a truncated version lacking 141 amino acids and thus the N-terminal AP2 domain; *ΔWRI1*) of the *Arabidopsis thaliana WRINKLED1* gene (*AtWRI1*; GenBank Accession No AY254038.2) were PCR-amplified from Arabidopsis seed cDNA and then fused downstream of the *HvSBEIIb* promoter from barley to form two different constructs, *i.e., *pSBE:*AtWRI1,* pSBE:*ΔAtWRI1* (Additional file 1: Fig. S1). Gene cassettes were inserted into the binary vector pCAMBIA1301. Vectors were transformed into the *Agrobacterium tumefaciens* strain EHA105, and then used for transformation of rice subspecies Indica and Japonica, as described [43]. In addition, *WRI1* and *ΔWRI1* were fused to the CaMV35S promoter (p35S) to form two constructs*;* p35S:*WRI1 and* p35S:*ΔWRI1*. The fusions were inserted into the binary vector pART27 [44], transformed into *Agrobacterium tumefaciens* strain AGL1 and used for for agro-infiltration of tobacco (*Nicotiana benthamiana*) leaves as described [45].

### RNA isolation and qRT-PCR analysis

All samples were harvested at the same time of the day (3 pm) and frozen in liquid nitrogen. Seed coat, endosperm and embryo from 9 daf, 11 daf and 14 daf developing seeds were separated in a cold room using a sharp scalpel and tweezer. Extraction of total RNA was done using Spectrum™ Plant Total RNA Kit (Sigma-Aldrich, US). Quantitative PCR analysis was performed as described [43, 46]. The oligonucleotides used for PCR in this paper are listed in Additional file 1: Table S3.

### Southern blot analysis

Southern blot analysis was performed as described before [32] with a small modification. Rice DNA was isolated by Dneasy® plant mini kit (Qiagen GmbH, Hilden, Germany) and digested by *Hind*III. The hygromycin-resistance gene *HPTII* was used as a probe for hybridization. Autoradiography was used for detection of hybridized bands.

### Total protein isolation and Western blot analysis

Total protein isolation and Western blot was performed as described [43, 46]. Equal loading was confirmed with the display of unified multiple bands (Leaf, stem: 31–52 kDa, seed: 12–17 kDa) on PAGE gel stained with Coomassie. First polyclonal antibodies to OsACC and OsPK-cyto from immunized rabbit were made based on the selected peptide sequences KMDPSRRAQLVEE and KDGKPIKLTKGQELT, respectively (BGI Genomics, Shenzhen, China). Anti-Rabbit lgG fragment Alkaline Phosphatase (Cat No. A3937, Sigma-Aldrich, US) was used as second antibody and Immuno-reacted bands were visualized in a BCIP/NBT (substrates for the phosphatase) solution for color development. Quantification of Western blot signals was performed with ImageJ (Additional file 1: Table S1).

### Fatty acid analysis

Analyses of fatty acids using TLC and GC were performed as described before [45, 47]. Briefly, different tissues were harvested at 3:00 pm, ground into fine powder in liquid nitrogen, and then used for homogenization in 3.75 ml of methanol/chloroform (2:1 v/v) and 1 ml 0.15 M acetic acid and extracted into chloroform [48]. For TAG analysis, the same amounts of extracts were loaded on a TLC plate, TAGs were separated with hexane:diethyl ether:acetic acid (70:30:1, v/v/v), visualized with primuline (Sigma-Aldrich) (0.01% primuline in acetone:water, 80:20), and the TLC area with TAG was removed and methylated. For assay of total fatty acids, extracts were used for direct methylation. 5 nmol of triheptadecanoin was added into samples as internal standard before methylation or loading on a TLC plate for total fatty acids or TAG analyses, respectively. Methylation was performed in situ with 2 ml of methanolic H_2_SO_4_ 2% (v/v) for 90 min at 90 °C. After extraction into hexane, the resulting fatty acid methyl esters were analyzed by GC. GC analyses were performed on a capillary column CP-Wax 58 (FFAP) CB, 50 m length, 0.32 mm i.d., and 0.20 μm film thickness (Varian Inc., CA, USA) for methyl esters on an Agilent GC 6890N equipped with an auto-sampler. Samples (1 µl) dissolved in hexane were injected at spitless mode. Helium was used as carrier gas at a column pressure of 15 psi, and nitrogen as make-up gas at a flow rate of 30 ml/min. The temperatures of injector and detector were 240 °C and 270 °C, respectively. Initial temperature was set to 100 °C for 1 min, and then temperature was raised to 250 °C at a rate of 10 °C/min for 10 min, and then held at 260 °C at a rate of 10 °C/min for 10 min.

### Carbohydrate analysis

Starch, glucose and sucrose were extracted and quantitated according to protocols provided in the kits from Megazyme (Bray, Co. Wicklow, Ireland) as described before [46].

### Transmission electron microscopy (TEM)

Seeds were harvested 14 daf at 3:00 pm, and then submerged in the fixative (4% paraformaldehyde + 2.5% glutaraldehyde in 0.1 M phosphate buffer, pH 7.2). Vacuum was performed to remove air from the seeds. The samples were fixed in the same solution with slow rotation for 12 h at room temperature. This was followed by 3 × 10 min washing in 100 mM phosphate buffer (pH 7.2) and the samples were stored at + 4 °C in buffer. Post-fixation was done in 2% osmium tetroxide in buffer overnight. The samples were then washed with buffer (3 × 10 min). Dehydration was performed in acidified 2,2-dimethoxypropane, followed by three acetone–Spurr epoxy series before being embedded in fresh Spurr epoxy resin and polymerized at 70 °C for 20 h. Sectioning, and preparation for TEM analyses were done as described [45, 49].

### Biochemical methane potential (BMP) analysis

The biochemical methane potential (BMP) of the rice straw and rice husks was determined using the commercial system AMPTS II (Bioprocess Control, Sweden) and all samples were analyzed in triplicate. Inoculum from all reactors collected from a biogas plant in Uppsala, Sweden, operating with mixed sewage sludge at wastewater treatment plant (WWTP). The inoculum:substrate ratio was set to 3:1 (VS basis) and the organic load was 3 g VS/L. The inoculum from WWTP had a total solids (TS) content of 3.1% of wet weight and a VS content of 2.0%. The TS and VS content was analyzed by drying according to APHA [50]. For the rice straw and rice husk powder, TS content were assumed to be the same as the dry weight. Dilution was made with tap water to final volume of 400 mL (flask volume 500 mL). Incubation temperature was set to 37 °C. To monitor background gas production from inoculum alone, three bottles were initiated by adding the same amount of inoculum and water to reach the same final liquid volume, but with no substrate. To confirm the activity of the inoculum, control bottles were also initiated with cellulose powder (Sigma-Aldrich) as substrate. The experiment ended when daily methane production fell below 1% of the accumulated methane production on a volume basis. The gas volumes produced were normalized to 1.01325 bar and temperature 273.2 K.

### Total organic carbon and nitrogen analysis

Harvest mature rice straw was dried for 3 days at 70 °C and then ground into fine powder for total organic carbon analysis. Mature rice grain was dried for 2 days at 45 °C, and then ground into fine powder after removing the husk. 0.5 g dry weight sample was used to perform further total nitrogen analysis. Total organic carbon and nitrogen analysis were performed in Agrilab AB (Sweden) based on the method of SS-ISO 10694 and SS-ISO 13878, respectively, with a slight modification. Samples were purged in the sealed purge chamber, after which gas was removed. The purged sample was transferred automatically to the furnace operated at 1100 °C. To ensure complete and rapid combustion (oxidation) of the sample, the furnace environment is composed of pure oxygen with a secondary oxygen flow directed to the sample via a ceramic lance. The combustion gases are swept from the furnace through a thermoelectric cooler to remove moisture and collected in thermostatically controlled ballast volume. Collected gases were equilibrated and mixed in the ballast before a representative aliquot of the gas was extracted and introduced into a flowing stream of inert gas for analysis. The aliquot gas is carried to non-dispersive infrared (NDIR) cells to detect carbon (as carbon dioxide). For total nitrogen analysis, the combustion process will convert any elemental nitrogen into N_2_ and NO_X_. Later steps will reduce NO_X_ to N_2_. A nitrogen result is eventually be produced from an output voltage from a TC cell, which has the N_2_ flow through it. Calibration was done by using substances with known carbon and nitrogen values. Control samples were used roughly every 5 samples.

### Combustion energy analysis

Lyophilized straw material was weighed (ca. 1.5 g DW), and the combustion energy was measured using an oxygen bomb calorimeter (Model 6400 isoperibol calorimeter; Parr Instruments, Moline, IL, USA). Ignition was under 25 atm O_2_ gas, analyses according to the supplier´s operating manual.

### Bioinformatics and statistical analysis

DNA sequences and PCR primers were analyzed and designed by the DNAstar® software (Madison, US). BLAST analyses were according to the NCBI website (http://blast.ncbi.nlm.nih.gove/). Statistical differences were analyzed by Student´s *t-*test.

## Supplementary Information

**Additional file 1: Figure S1.** Primary structure of the *Arabidopsis thaliana* WRINKLED 1 protein. **Figure S2**. Southern blot analysis of WRI1 and ΔWRI1 Indica rice plants. **Figure S3**. TAG levels in embryo-removed mature seed of wild-type Indica rice, and *AtWRI1* transformants. **Figure S4**. Phenotypic characteristics of homozygous WRI1 Indica rice plants compared with wild-type MH86. **Figure S5**. Phenotypic characteristics of homozygous WRI1 Japonica rice plants compared with wild-type Nipponbare. **Figure S6**. Effect of *AtWRI1* on gene expression in vegetative tissues of Nipp and full-length *AtWRI1* transformants. **Figure S7**. Fatty acid levels in leaf and stem of Nipp and full-length *AtWRI1* transformants. **Figure S8**. Fatty acid-related gene expression in reproductive tissues of Nipp and full-length *AtWRI1* transformants. **Figure S9**. Fatty acid and starch levels in reproductive tissues of Nipp and full-length *AtWRI1* transformants. **Figure S10.** Total nitrogen value in mature husk-removed rice grain from wild-type Indica rice, and full-length *AtWRI1* transformants. **Figure S11.** Total organic carbon and combustion energy values of straw from wild-type Indica rice plants, and full-length *AtWRI1* transformants. **Table S1**. Quantitation of Western blot analyses. **Table S2**. Summary of biomethane yield from different rice straw and their oil, carbohydrate and total organic carbon contents. **Table S3**. Summary of oligonucleotides used in this investigation.

## Data Availability

All data of this study are included in the published article and its supplemental files.

## Abbreviations

WRI1: WRINKLED1
TAG: Triacylglycerol
SBEIIb: Starch branching enzyme IIb
Os*PK-cyto*: Pyruvate kinase cytosolic isozyme
Os*PK-chlor*: Pyruvate kinase isozyme g chloroplast
Os*ACC*: Acetyl-CoA carboxylase
Os*DGAT1*: Diacylglycerol transferase 1
daf: Day after fertilization
FW: Fresh weight
DW: Dry weight
Nml: Normal ml
TS: Total solids
Nipp: Nipponbare. BMP: Biochemical methane potential
WWTP: Wastewater treatment plant
