# Supplementary material for: Improved bioenergy value of residual rice straw by increased lipid levels from upregulation of fatty acid biosynthesis
Source: Biotechnol Biofuels Bioprod. 2023 May 27;16:90. doi: 10.1186/s13068-023-02342-y (PMC10224602; doi:10.1186/s13068-023-02342-y)
Supplement: Supplementary file 1 — Additional file 1: Figure S1. Primary structure of the Arabidopsis thaliana WRINKLED 1 protein. Figure S2. Southern blot analysis of WRI1 and ΔWRI1 Indica rice plants. Figure S3. TAG levels in embryo-removed mature seed of wild-type Indica rice, and AtWRI1 transformants. Figure S4. Phenotypic characteristics of homozygous WRI1 Indica rice plants compared with wild-type MH86. Figure S5. Phenotypic characteristics of homozygous WRI1 Japonica rice plants compared with wild-type Nipponbare. Figure S6. Effect of AtWRI1 on gene expression in vegetative tissues of Nipp and full-length AtWRI1 transformants. Figure S7. Fatty acid levels in leaf and stem of Nipp and full-length AtWRI1 transformants. Figure S8. Fatty acid-related gene expression in reproductive tissues of Nipp and full-length AtWRI1 transformants. Figure S9. Fatty acid and starch levels in reproductive tissues of Nipp and full-length AtWRI1 transformants. Figure S10. Total nitrogen value in mature husk-removed rice grain from wild-type Indica rice, and full-length AtWRI1 transformants. Figure S11. Total organic carbon and combustion energy values of straw from wild-type Indica rice plants, and full-length AtWRI1 transformants. Table S1. Quantitation of Western blot analyses. Table S2. Summary of biomethane yield from different rice straw and their oil, carbohydrate and total organic carbon contents. Table S3. Summary of oligonucleotides used in this investigation. [file 13068_2023_2342_MOESM1_ESM.docx]

Supporting Information for:

**Improved bioenergy value of residual rice straw by increased lipid levels from up-regulation of fatty acid biosynthesis**

Yunkai Jin^1§^, Jia Hu^1§^, Jun Su^1,2^, Selcuk Aslan^1^, Yan Lin^2^, Lu Jin^1,3^, Simon Isaksson^4^, Chunlin Liu^3^, Feng Wang^2^, Anna Schnürer^4^, Folke Sitbon^1*^, Per Hofvander^5^, Chuanxin Sun^1^*

1, Department of Plant Biology, Swedish University of Agricultural Sciences, The Linnean Centre for Plant Biology, Box 7080, SE-75007 Uppsala, Sweden

2, Institute of Biotechnology, Fujian Academy of Agricultural Sciences, Fuzhou 350003, China

3, Hunan Provincial Key Laboratory of Crop Germplasm Innovation and Utilization, Hunan Agricultural University, Changsha 410128, China

4, Department of Molecular Sciences, Swedish University of Agricultural Sciences, Box 7015, SE-750 07 Uppsala, Sweden

5, Department of Plant Breeding, Swedish University of Agricultural Sciences, P.O. Box 190, SE-23422 Lomma, Sweden

§ Equal contributors

* Shared Authors for Correspondence: Folke Sitbon; Chuanxin Sun

Department of Plant Biology, Swedish University of Agricultural Sciences, The Linnean Centre for Plant Biology, Box 7080, SE-75007 Uppsala, Sweden

Email: [Chuanxin.Sun@slu.se](mailto:Chuanxin.Sun@slu.se); Phone: +46-18-673252

Email: [Folke.Sitbon@slu.se](mailto:Folke.Sitbon@slu.se); Phone: +46-18-673243

**ORCID**

Chuanxin Sun, <https://orcid.org/0000-0003-2755-0443>

Folke Sitbon, <https://orcid.org/0000-0002-6852-069X>

**Contents**

Fig. S1 – S11

Table S1 – S3

**Supplementary Figures**


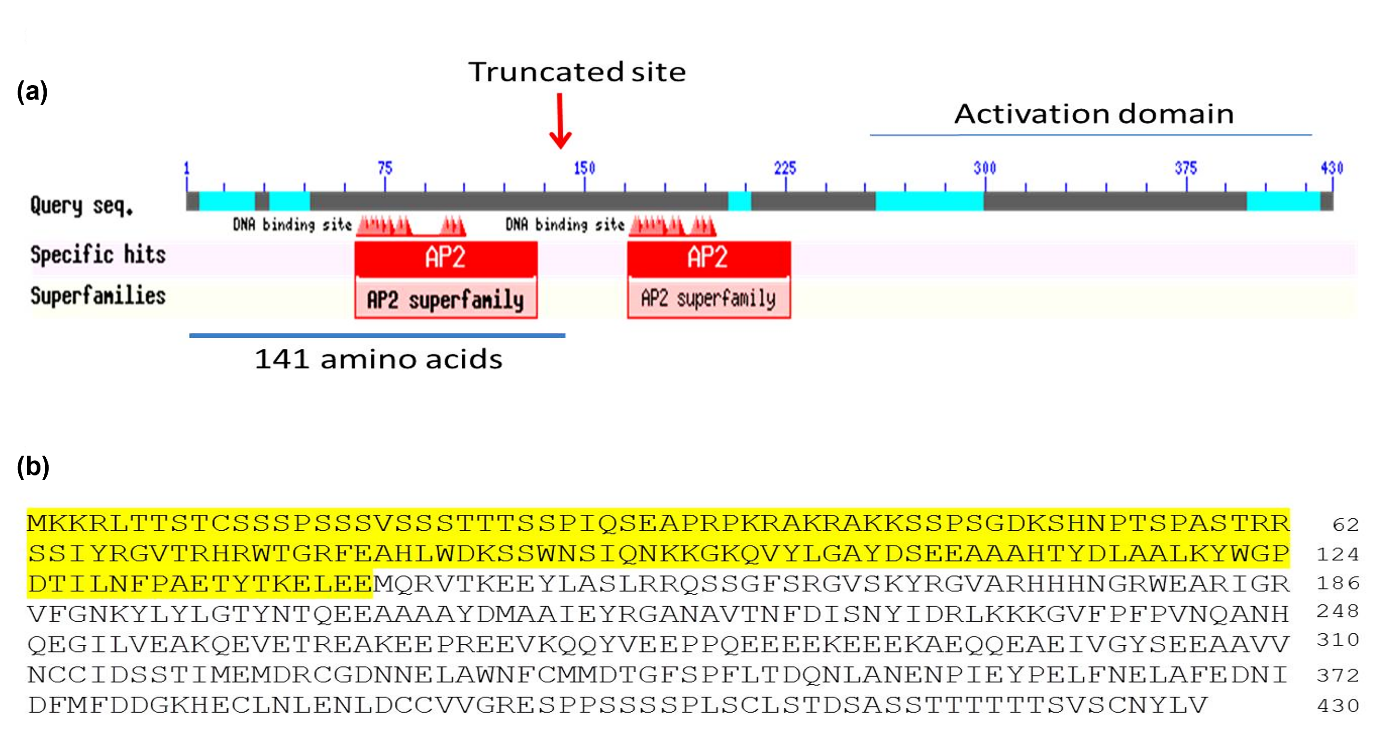


**Fig. S1** Primary structure of the *Arabidopsis thaliana* WRINKLED 1 (WRI1) protein. (**a**) Schematic representation of Arabidopsis WRI1 protein as a member of the plant AP2/ERF transcription factor superfamily. The site for generation of the truncated ΔWRI1 protein used in this study is arrowed, and a putative activation domain is indicated. (**b**) Amino acid sequence of Arabidopsis WRI1 protein. The 114 amino acids deleted in the truncated ΔWRI1 protein are highlighted in yellow.


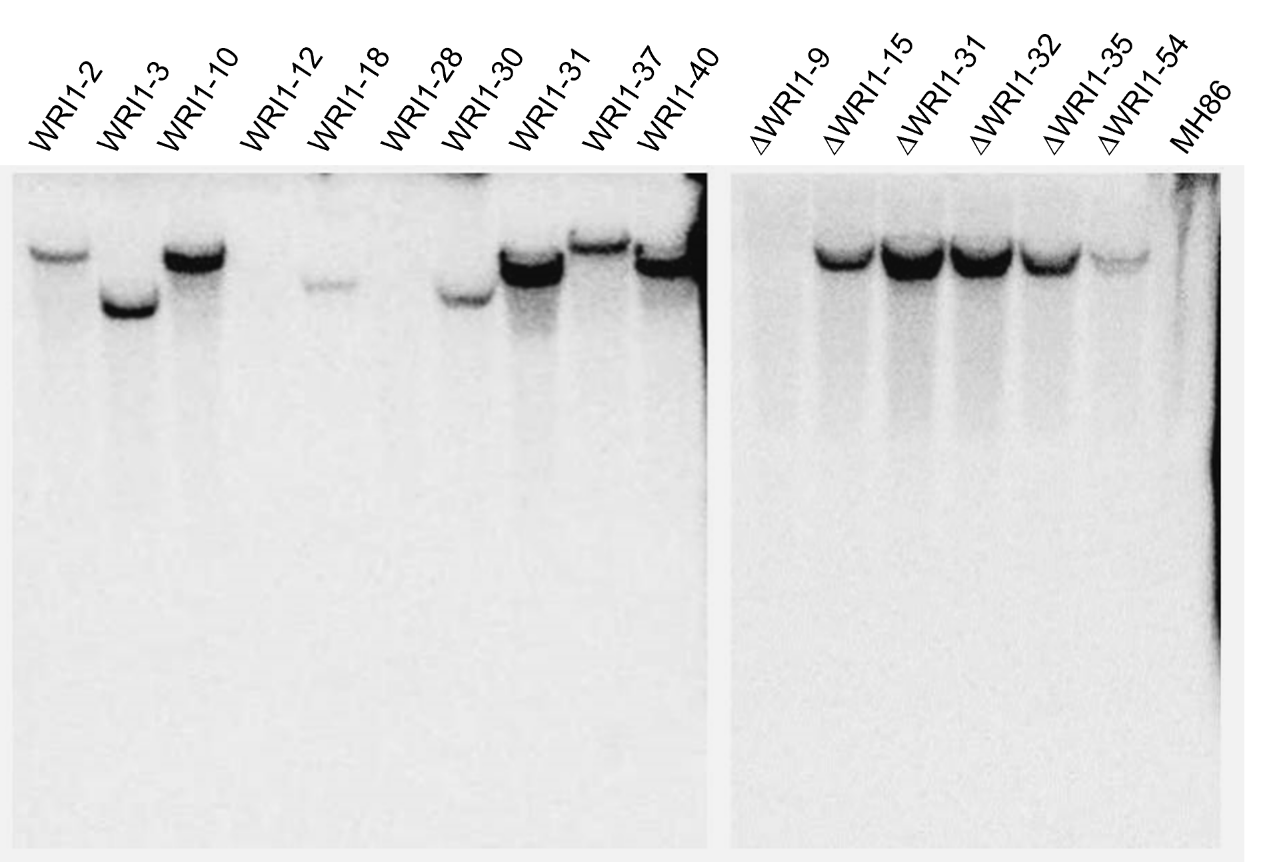


**Fig. S2** Southern blot analysis of WRI1 and ΔWRI1 Indica rice transformants. Total DNA was digested by *Hind*III and used for Southern blot analysis using the T-DNA hygromycin phosphotransferase gene (*HPT II*) as a labelled probe. Wild-type Minghui 86 (MH86) DNA was used as a negative control. T_2_ or T_4_ generations were used for WRI1 and ΔWRI1 rice plants, respectively.


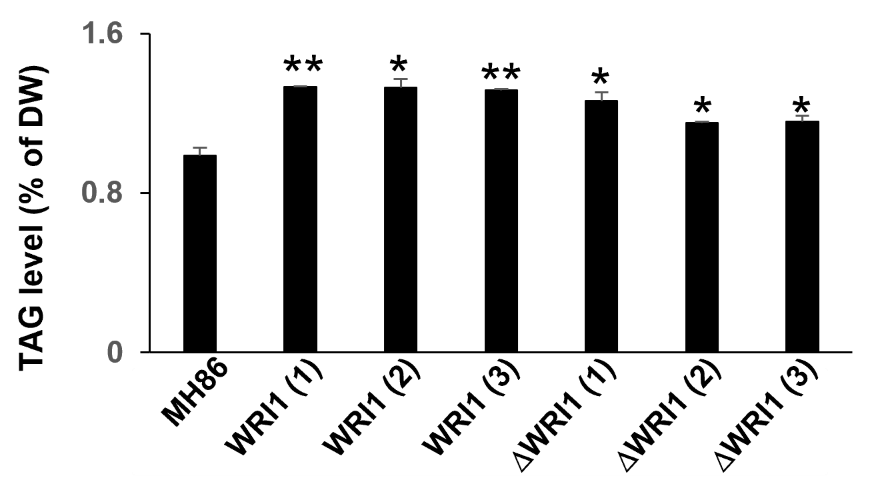


**Fig. S3** TAG levels in embryo-removed mature seed of wild-type Indica rice Minghui 86 (MH86), and derived WRI1 transformants. TAG levels were determined using gas chromatography. Mean value ± SD from three biological replicates. Numbers in parenthesis indicate different lines. DW, dry weight; WRI1, full-length *AtWRI1* transformants; ΔWRI1, truncated *AtWRI1* transformants. Asterisks indicate a statistical difference compared to the wild type, significant at p≤ 0.05 (*) or p≤ 0.01 (**) (Student´s *t*-test).

**
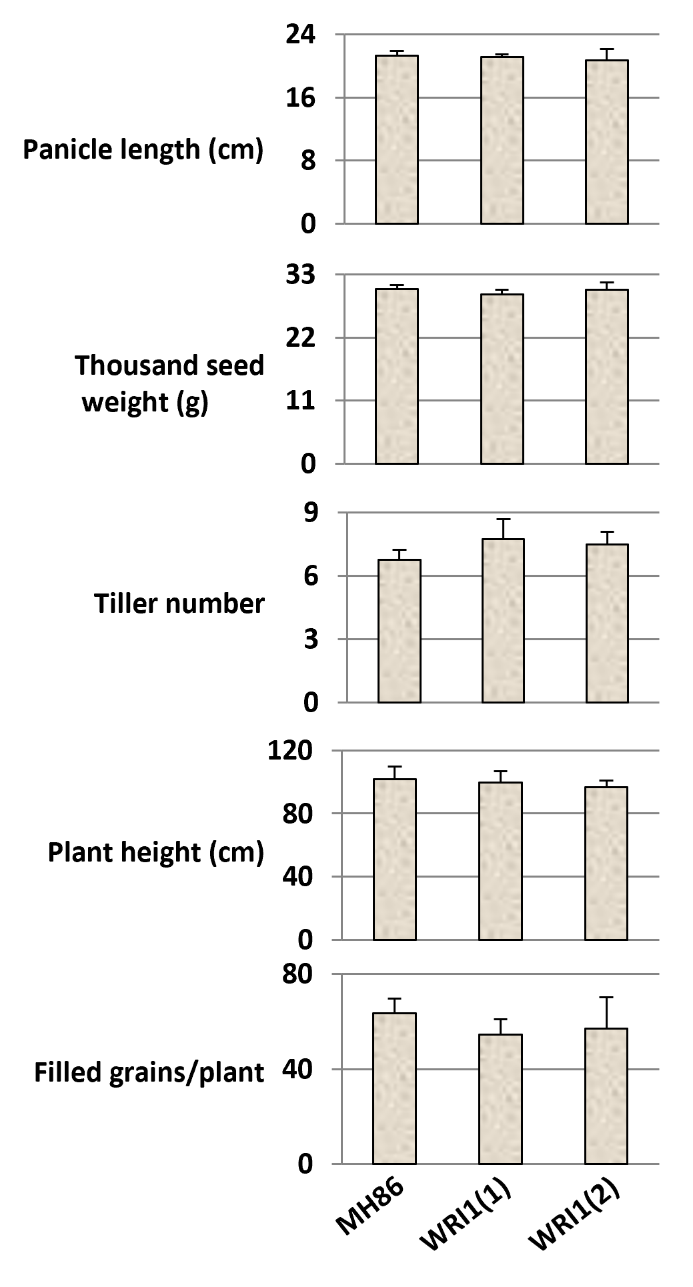
**

**Fig. S4** Phenotypic characteristics of Indica rice Minghui 86 (MH86) plants, and derived homozygous WRI1 transformants. Plants were grown under controlled conditions in a phytotron, and panicle length, thousand kernel weight, tiller number, plant height, and filled grains per plant were measured. WRI1, full-length *AtWRI1* transformants. WRI1(1) and WRI1(2) indicate different transgenic lines. Mean value ± SD from five biological replicates for each genotype. No differences between genotypes were statistically significant (Student´s *t*-test).


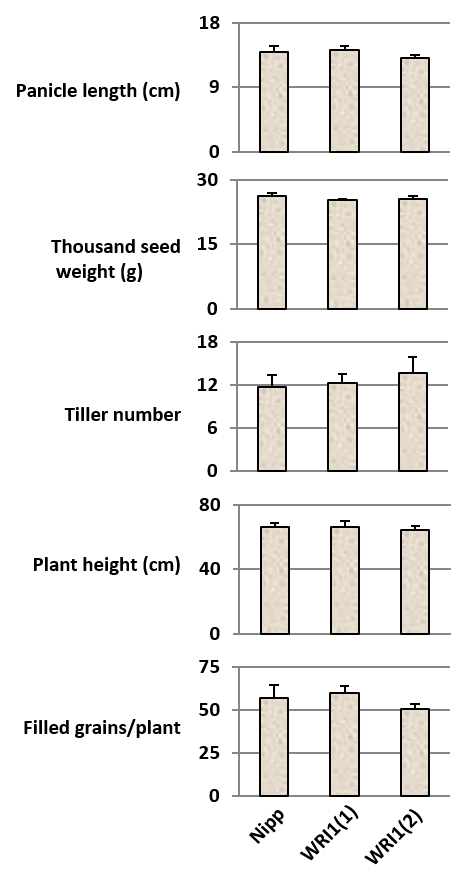


**Fig. S5** Phenotypic characteristics of Japonica rice Nipponbare (Nipp) plants, and derived homozygous WRI1 transformants. Plants were grown under controlled conditions in a phytotron, and panicle length, thousand kernel weight, tiller number, plant height, and filled grains per plant were measured. WRI1: full-length *AtWRI1* transformants. WRI1(1) and WRI1(2) indicate different transgenic lines. Mean value ± SD from five biological replicates for each genotype. No differences between genotypes were statistically significant (Student´s *t*-test).


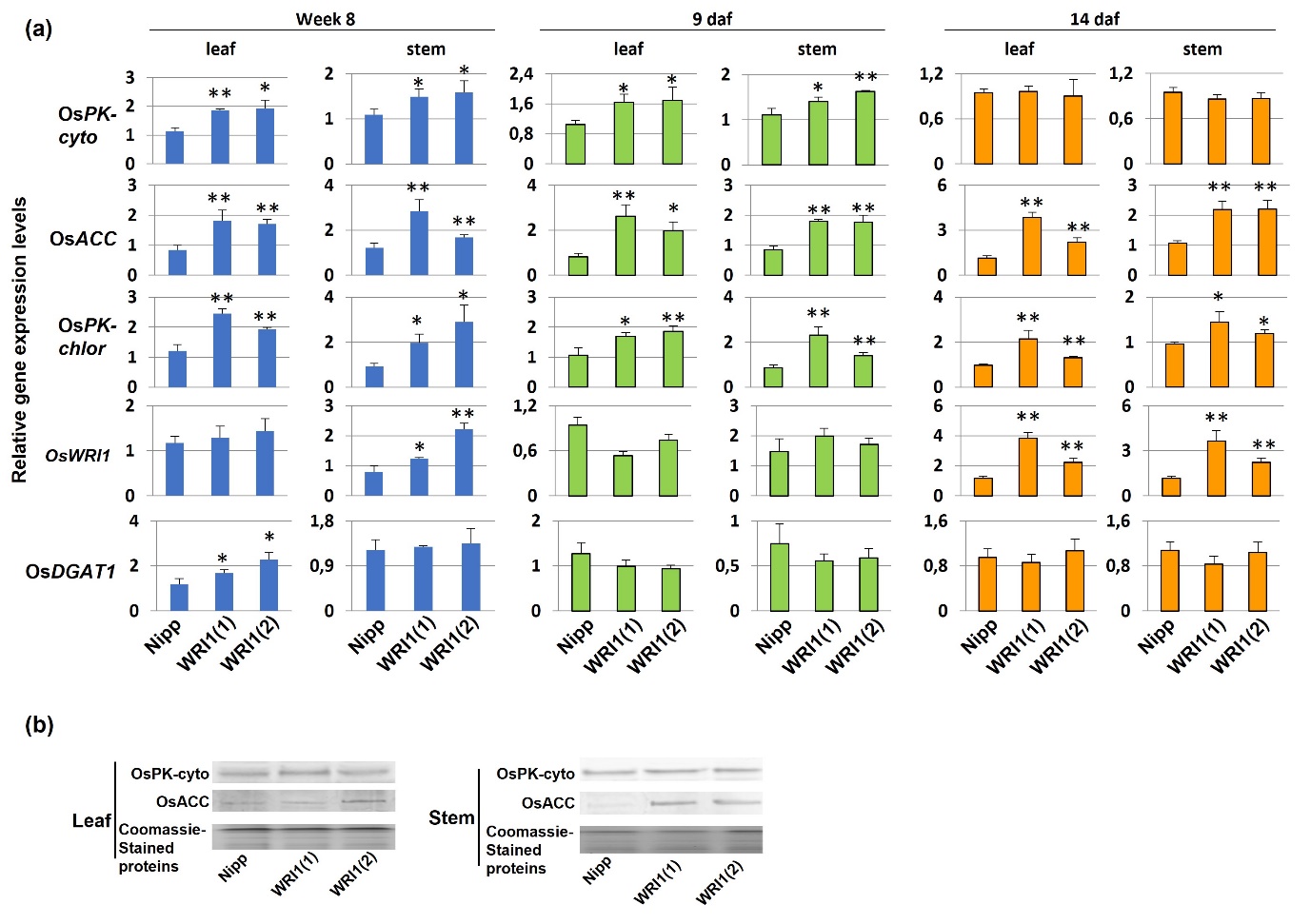


**Fig. S6** Effect of AtWRI1 on gene expression in vegetative tissues of Japonica rice Nipponbare (Nipp) plants, and derived full-length At*WRI1* transformants. (**a**) Relative expression in leaf and stem of genes in fatty acid synthesis, as analyzed by qRT-PCR at different times after fertilization (daf). Mean value ± SD from three biological replicates for each genotype. Numbers in parenthesis indicate different lines. Asterisks indicate a statistical difference compared to the wild type, significant at p≤ 0.05 (*) or p≤ 0.01 (**) (Student´s *t*-test). (**b**) Western blot analysis of OsPK-cyto and OsACC protein levels in leaves and stems in Nipp and two transgenic lines. WRI1, full-length *AtWRI1* transformants. WRI1(1) and WRI1(2) indicate different transgenic lines. Quantification of signal intensities are shown in Table S2.


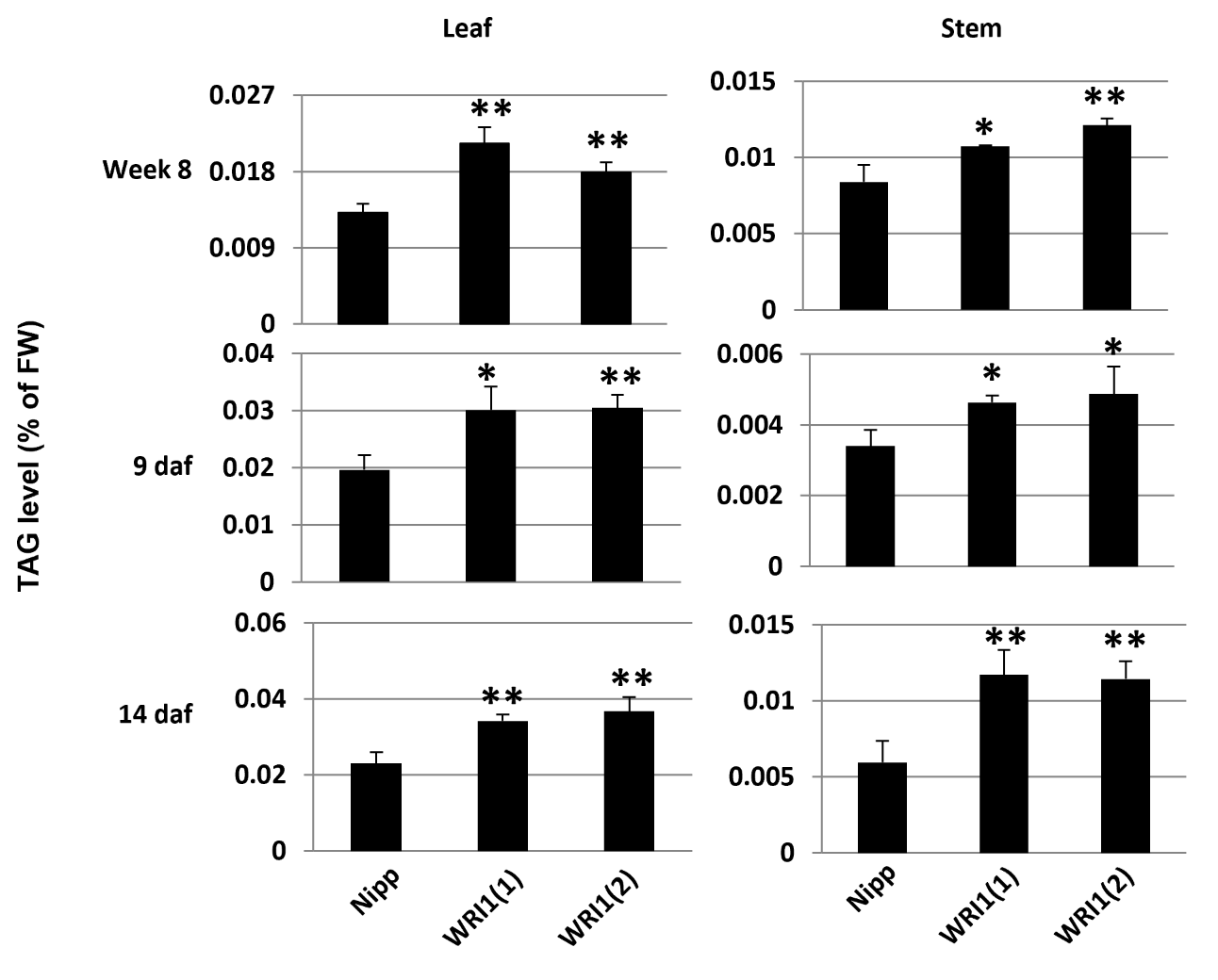


**Fig. S7** Fatty acid levels in leaf and stem of Japonica rice Nipponbare (Nipp), and derived full-length *AtWRI1* transformants. Gas chromatography quantification of total TAG levels in leafs and stems measured at week 8, and at 9 or 14 days after fertilization (daf), in Nipp and two transgenic lines. Numbers in parenthesis indicate different lines. Mean value ± SD from three biological replicates for each line. FW, fresh weight; WRI1, full-length *AtWRI1* transformants. WRI1(1) and WRI1(2) indicate different transgenic lines. Asterisks indicate a statistical difference compared to the wild type, significant at p≤ 0.05 (*) or, p≤ 0.01 (**) (Student´s *t*-test).


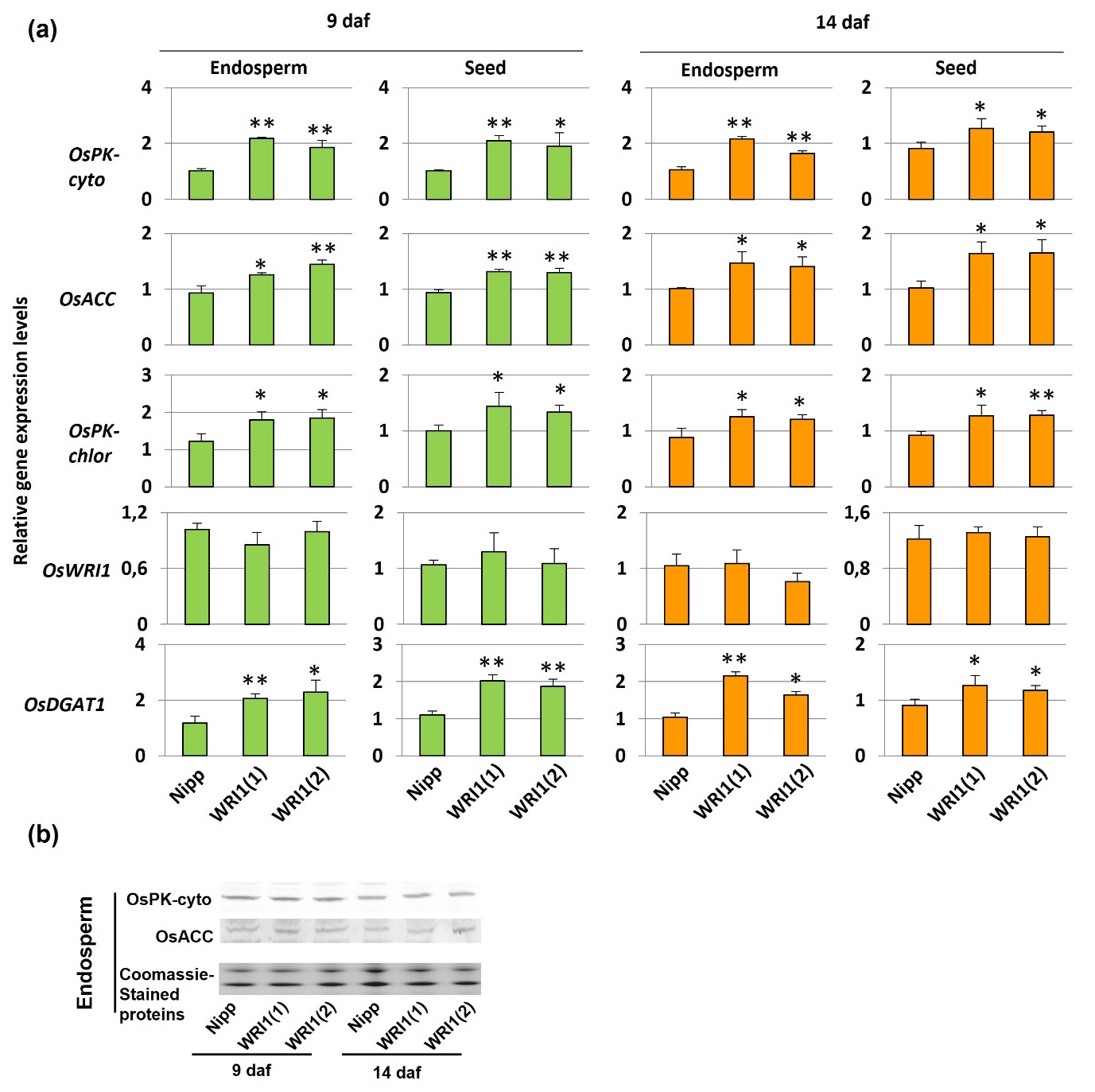


**Fig. S8** Fatty acid-related gene expression in reproductive tissues of Japonica rice Nipponbare (Nipp), and derived full-length At*WRI1* transformants. (**a**) Relative gene expression in endosperm and seed at different times after fertilization (daf). Mean value ± SD from three biological replicates for each genotype. Asterisks indicate a statistical difference compared to the wild type, significant at p≤ 0.05 (*), or p≤ 0.01 (**) (Student´s *t*-test). (**b**) Western blot analysis of OsPK-cyto and OsACC protein levels in endosperm at 14 daf in Nipp and two transgenic lines. Quantification of signal intensities are shown in Table S2. WRI1, full-length *AtWRI1* transformants. WRI1(1) and WRI1(2) indicate different transgenic lines.


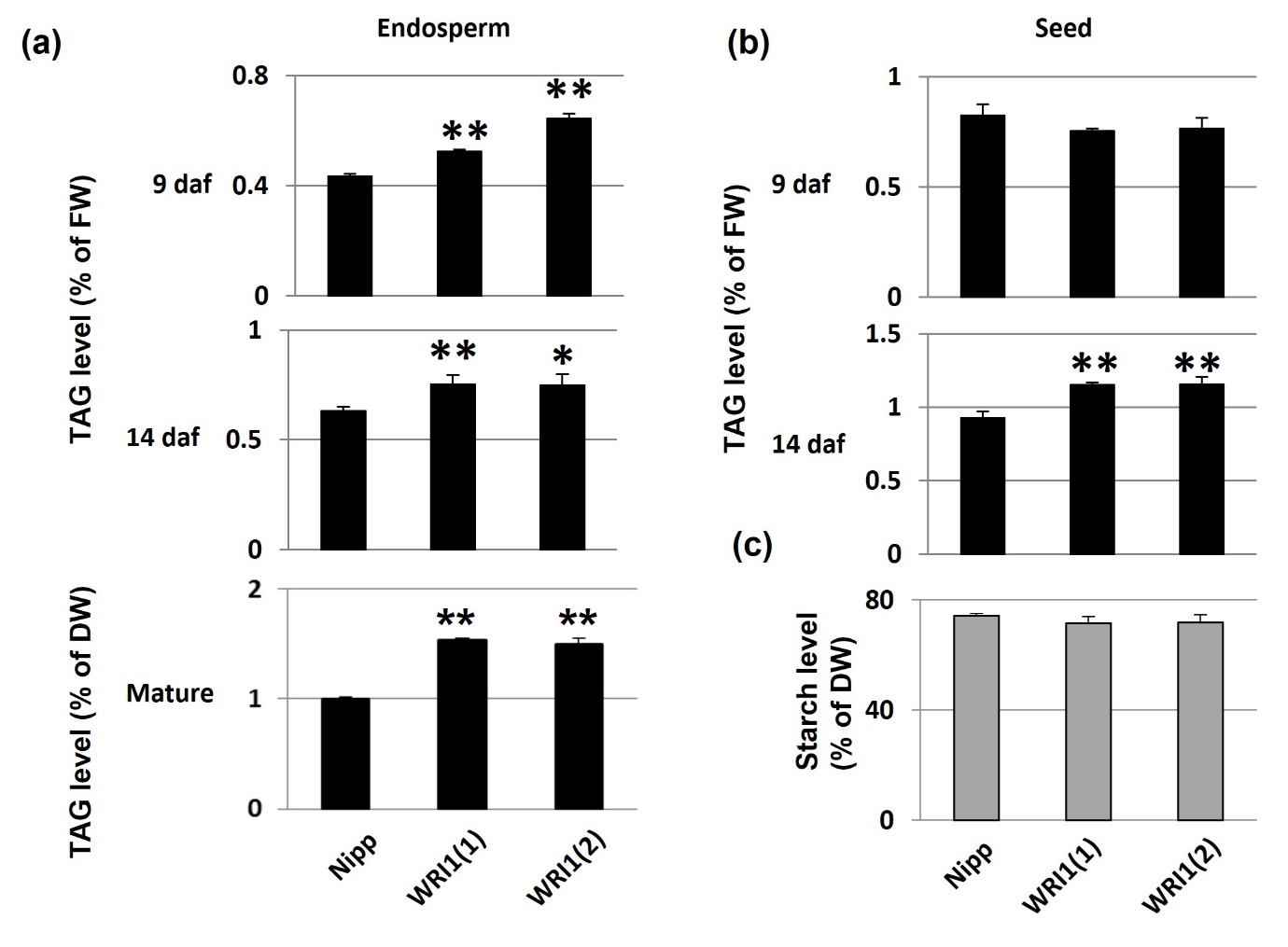
**Fig. S9** Fatty acid and starch levels in reproductive tissues of Japonica rice Nipponbare (Nipp), and derived full-length At*WRI1* transformants. (**a**) Quantification by gas chromatography analysis of total TAG levels in endosperm, and mature seeds without embryo. (**b**) Total TAG level in whole seeds. (**c**) Starch level in mature seeds without embryo. Mean value ± SD from three biological replicates for each genotype. DW, dry weight; FW, fresh weight; WRI1, full-length *AtWRI1* transformants. WRI1(1) and WRI1(2) indicate different transgenic lines. Asterisks indicate a statistical difference compared to the wild type, significant at p≤ 0.05 (*), or p≤ 0.01 (**) (Student´s *t*-test).

**
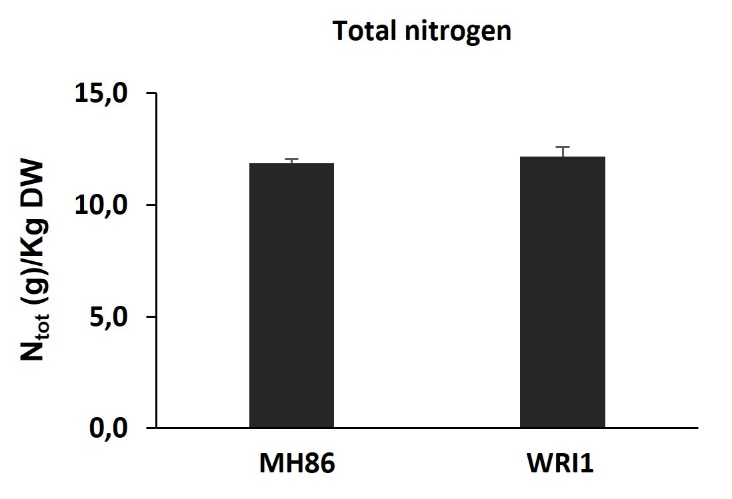
**

**Fig. S10** Level of total nitrogen in husk-removed rice seeds from wild-type Indica rice plants Minghui 86 (MH86), and derived full-length *AtWRI1* transformants (WRI1). Mean value ± SD from three biological replicates for MH86, and mean value ± range of triplicate analyses of two different lines for WRI1 rice. N_tot_, total nitrogen; DW, dry weight. Differences between genotypes were not statistically significant (Students *t*-test).


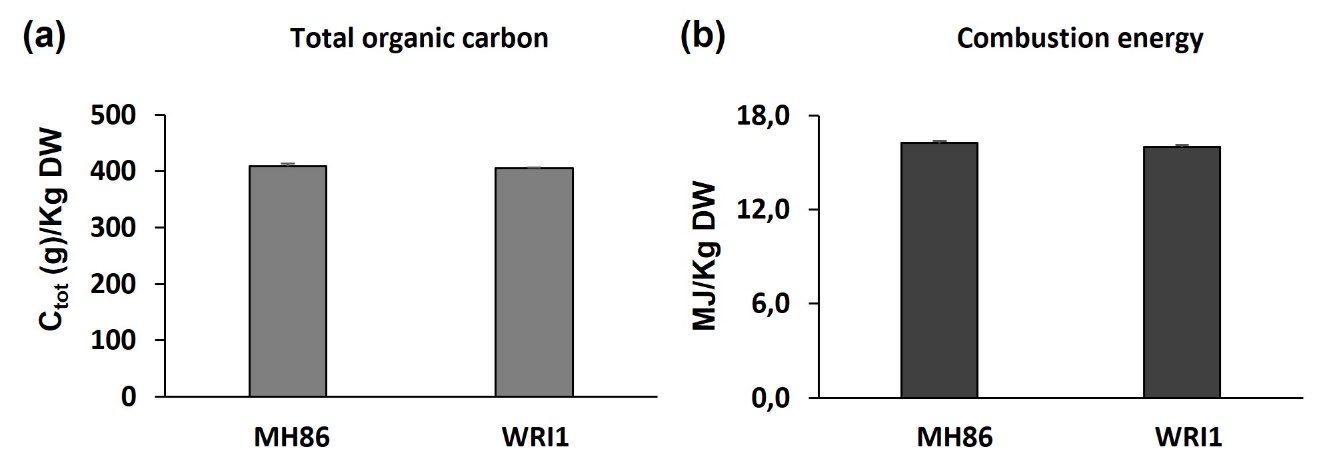
**Fig. S11** Level of total organic carbon (a) and combustion energy (b) in straw from wild-type Indica rice plants Minghui 86 (MH86), and derived full-length *AtWRI1* transformants (WRI1). Mean value ± SD from three biological replicates for MH86, and mean value ± range of triplicate analyses of two different lines for WRI1 transformants. C_tot_, total organic carbon; DW, dry weight. Differences between genotypes were not statistically significant (Students *t*-test).

**Tables**

| **Table S1.** Quantitation of Western blot analyses of wild-type Indica rice (MH86) and Japonica rice (Nipp.), and the derived full-length (*WRI1*) and truncated (*ΔWRI1*) *AtWRI1* transformants. Band signal intensities from digital reproductions of the original Western blots using antibodies against the rice proteins pyruvate kinase cytosolic isozyme (OsPK-cyto), and Acetyl-CoA carboxylase (OsACC), were quantified digitally using the ImageJ software, and are expressed as relative units. Numbers in parenthesis indicate different lines. daf: days after fertilization. | | | | | | | | | | | | | | | | | | | | | | | | |
| --- | --- | --- | --- | --- | --- | --- | --- | --- | --- | --- | --- | --- | --- | --- | --- | --- | --- | --- | --- | --- | --- | --- | --- | --- |
| **Fig. 2C** | | |  | | | | MH86 | | | | WRI1 | | | | | | | | | ΔWRI1 | | | | |
| Tissue | | | Protein | | | |  | | | | Line 1 | | | | Line 2 | | | | | Line 1 | | | Line 2 | |
| Leaf | | | OsPK-cyto | | | | 0.093 | | | | 0.113 | | | | 0.114 | | | | | 0.118 | | | 0.112 | |
|  |  |  | OsACC | | | | 0.062 | | | | 0.081 | | | | 0.067 | | | | | 0.109 | | | 0.130 | |
|  |  |  | Coomassie | | | | 0.502 | | | | 0.457 | | | | 0.491 | | | | | 0.478 | | | 0.402 | |
| Stem | | | OsPK-cyto | | | | 0.08 | | | | 0.104 | | | | 0.099 | | | | | 0.087 | | | 0.112 | |
|  |  |  | OsACC | | | | 0.078 | | | | 0.120 | | | | 0.132 | | | | | 0.107 | | | 0.113 | |
|  |  |  | Coomassie | | | | 0.211 | | | | 0.185 | | | | 0.186 | | | | | 0.185 | | | 0.163 | |
| **Fig. 4C** | 9 daf | | | | | | | | 11 daf | | | | | | | | 14 daf | | | | | | | |
| OsPK-cyto | 0.049 | | | | 0.063 | | 0.091 | | 0.062 | | | | 0.066 | | | 0.07 | 0.036 | | | | 0.062 | | | 0.05 |
| OsACC | 0.061 | | | | 0.076 | | 0.102 | | 0.073 | | | | 0.06 | | | 0.058 | 0.047 | | | | 0.064 | | | 0.06 |
| Coomassie | 0.181 | | | | 0.196 | | 0.162 | | 0.160 | | | | 0.169 | | | 0.176 | 0.169 | | | | 0.181 | | | 0.154 |
| **Fig. S6B** | | | | Leaf | | | | | | | | | | | Stem | | | | | | | | | |
|  | | | | Nipp | | | | WRI1(1) | | | | WRI1(2) | | | Nipp | | | WRI1(1) | | | | WRI1(2) | | |
| OsPK-cyto | | | | 0.126 | | | | 0.132 | | | | 0.121 | | | 0.076 | | | 0.082 | | | | 0.079 | | |
| OsACC | | | | 0.154 | | | | 0.155 | | | | 0.164 | | | 0.039 | | | 0.095 | | | | 0.076 | | |
| Coomassie | | | | 0.355 | | | | 0.340 | | | | 0.353 | | | 0.281 | | | 0.263 | | | | 0.311 | | |
| **Fig. S8B** | | 9 daf | | | | | | | | | | | | 14 daf | | | | | | | | | | |
|  | | Nipp | | | | WRI1(1) | | | | WRI1(2) | | | | Nipp | | | | | WRI1(1) | | | WRI1(2) | | |
| OsPK-cyto | | 0.077 | | | | 0.081 | | | | 0.078 | | | | 0.06 | | | | | 0.065 | | | 0.06 | | |
| OsACC | | 0.092 | | | | 0.109 | | | | 0.094 | | | | 0.07 | | | | | 0.071 | | | 0.094 | | |
| Coomassie | | 0.542 | | | | 0.549 | | | | 0.552 | | | | 0.568 | | | | | 0.499 | | | 0.436 | | |

**Table S2.** Summary of biomethane yield from different rice straw and their oil, carbohydrate and total organic carbon contents

| Genotyping | Bio-methane yield (Nml gTS^-1^) | TAG  (g/kg DW) | Total fatty acids (g/kg DW) | Carbohydrate (g/kg DW) | Total organic carbon (g/kg DW) |
| --- | --- | --- | --- | --- | --- |
| MH86 | 144.6±7.9 | 0.183±0.017 | 0.598±0.027 | 99.1±5.6 | 408±4.9 |
| WRI1 rice | 172.8±12.6* | 0.402±0.022** | 0.990±0.082** | 102.3±4.0 | 405±1.0 |

Mean value ± SD from three biological replicates for each genotype. Asterisks indicate a statistical difference compared to the wild type, significant at p≤ 0.05 (*) or p≤ 0.01 (**) (Student´s *t*-test). Nml: Normal ml; TS, Total solids; TAG, triacylglycerol; DW, dry weight.

| **Table S3.** Summary of oligonucleotides used in this investigation. | | | | |
| --- | --- | --- | --- | --- |
| Primer name | Gene | GenBank Acc No | Primer sequence (5'→3') | Used for |
| OsActin11Fwd | *ACTIN-1* | KC140126 | GCATCTCTCAGCACATTCCA | qPCR |
| OsActin11Rev |  |  | GCGATAACAGCTCCTCTTGG |  |
| Atwri1Fwd-1 | *AtWRI1* | AY254038.2 | CGTGGTGGGAAGAGAGAGC | qPCR |
| Atwri1Rev-1 |  |  | TGTTGATGAAGCAGAGTCAGTAGA |  |
| Atwri1Fwd-2 | *AtWRI1* | AY254038.2 | ATGGAAATGGATCGTTGTGG | RT-PCR |
| Atwri1Rev-2 |  |  | AGAGGGTGGGCTCTCTCTTC |  |
| Atwri1Fwd-3 | At*WRI1* | AY254038.2 | AAACCCGGGTAATGAAGAAGCGCTTAACC | cDNA cloning |
| Atwri1Rev-3 |  |  | AAAGAGCTCTCAGACCAAATAGTTACAA |  |
| QPKcyto-1Fwd | Os*PK-cytol* | EU267984 | TTCTGCCAAAGCCACCGATTC | qPCR |
| QPKcyto-1Rev |  |  | ACGGATGCGACGCCAATACG |  |
| QACC-1Fwd | Os*ACC* | [NC_008398](http://www.ncbi.nlm.nih.gov/nuccore/NC_008398) | GTCTCCGCGGATCCACAGTATAC | qPCR |
| QACC-1Rev |  |  | ATTTATTTCACATACAGACGCATTT |  |
| QPKchlo-1Fwd | Os*PK-chlor* | NM_001071986 | AGGAGGGGAGTCTGCCTGTGAG | qPCR |
| QPKchlo-1Rev |  |  | TGAGATAAACAAAAAGAAAGTCGC |  |
| OsWRI1F | Os*WRI1* | Os11g0129700 | GGCATATAGCAACTAGTTGT | qPCR |
| OsWRI1R |  |  | CTAGTGGTAAGTTGGCAAAC |  |
| OsDGAT1 FP | Os*DGAT1* | XM_015783920 | TCCGTCGCAGGATCCGCTCG | qPCR |
| OsDGAT1 RP |  |  | CGTTGGAGTCGCCGCCACGG |  |
